# PPE38-Secretion-Dependent Proteins of *M. tuberculosis* Alter NF-kB Signalling and Inflammatory Responses in Macrophages

**DOI:** 10.3389/fimmu.2021.702359

**Published:** 2021-07-02

**Authors:** James Gallant, Tiaan Heunis, Caroline Beltran, Karin Schildermans, Sven Bruijns, Inge Mertens, Wilbert Bitter, Samantha L. Sampson

**Affiliations:** ^1^ Department of Science and Technology/National Research Foundation Centre of Excellence in Biomedical Tuberculosis Research, South African Medical Research Council Centre for Tuberculosis Research, Division of Molecular Biology and Human Genetics, Department of Biomedical Sciences, Faculty of Medicine and Health Sciences, Stellenbosch University, Cape Town, South Africa; ^2^ Section Molecular Microbiology, Amsterdam Institute for Molecules, Medicines and Systems, Vrije Universiteit Amsterdam, Amsterdam, Netherlands; ^3^ Sir William Dunn School of Pathology, University of Oxford, Oxford, United Kingdom; ^4^ Health Unit, VITO, Antwerp, Belgium; ^5^ Department of Molecular Cell Biology and Immunology, Cancer Center Amsterdam, Amsterdam Infection and Immunity Institute, Vrije Universiteit Amsterdam, Amsterdam UMC, Amsterdam, Netherlands; ^6^ Medical Microbiology and Infection Control, Vrije Universiteit Amsterdam, Amsterdam UMC, Amsterdam, Netherlands

**Keywords:** *Mycobacterium tuberculosis*, proteomics, NF-KB signalling, PE/PPE, macrophage

## Abstract

It was previously shown that secretion of PE-PGRS and PPE-MPTR proteins is abolished in clinical *M. tuberculosis* isolates with a deletion in the *ppe38-71* operon, which is associated with increased virulence. Here we investigate the proteins dependent on PPE38 for their secretion and their role in the innate immune response using temporal proteomics and protein turnover analysis in a macrophage infection model. A decreased pro-inflammatory response was observed in macrophages infected with PPE38-deficient *M. tuberculosis* CDC1551 as compared to wild type bacteria. We could show that dampening of the pro-inflammatory response is associated with activation of a RelB/p50 pathway, while the canonical inflammatory pathway is active during infection with wild type *M. tuberculosis* CDC1551. These results indicate a molecular mechanism by which *M. tuberculosis* PE/PPE proteins controlled by PPE38 have an effect on modulating macrophage responses through NF-kB signalling.

## Introduction

*Mycobacterium tuberculosis* is an important human pathogen that has adapted to survive and replicate within human macrophages (1). This lifestyle requires the presence of virulence factors that have evolved to enable intracellular growth. One strategy to identify virulence factors is by comparing the genomes of pathogenic and non-pathogenic mycobacteria. The most striking result of such an analysis is a large number of genes encoding PE and PPE proteins in *M. tuberculosis* compared to *M. smegmatis* (2). The PE and PPE proteins belong to two unique but related protein families and are known to be secreted to the cell surface or extracellular milieu through the type VII secretion system (3). The type VII secretion system is further represented by five loci in *M. tuberculosis*, named ESX-1 to ESX-5 (4). Of these five loci, ESX-2 and ESX-5 are the most recent, with ESX-5 being associated with the evolutionary split of the fast- and slow-growing mycobacteria (5). The PE/PPE proteins are characterised by the presence of a conserved N-terminal domain, including a Proline-Glutamic acid (PE) or a Proline-Proline-Glutamic acid (PPE) conserved motif (PPE) conserved motif (2). There are approximately 100 genes in *M. tuberculosis* coding for PE proteins and the major sub-family is classified as the PE-PGRS proteins, due to a large number of GC repeats in the genes encoding them (Polymorphic GC-Rich Sequence). This sub-family is characterised by multiple Gly-Gly-Ala/Gly-Gly-Asn repeats in the C-terminal region (6, 7). The PE-PGRS proteins are unique to slow growing mycobacteria, localised to the cell surface and secreted through the ESX-5 system (4, 8–11). Furthermore, recent studies demonstrated that the C-terminal region of PE-PGRS proteins can be cleaved from the PE domain and can be released to interact with the host (12). The largest subfamily of PPE proteins are the PPE-MPTR proteins (Major Polymorphic Tandem Repeat), which are also specific to slow-growing mycobacteria and secreted *via* ESX-5 (3). Taken together, the PE-PGRS and PE-MPTR proteins are interesting candidates for studying host-pathogen interactions considering their evolutionary history and extracellular localisation. However, elucidating the effects of PE-PGRS and PPE-MPTR proteins is hampered by the large number of proteins present in these groups. Recently, it has been shown that the *ppe38-71* operon, specifically the PPE38 protein, is involved in mediating secretion of both these important subfamilies and when deleted detectable secretion is abolished (13). More recent follow up studies conducted in *M. africanum* and *M. microti* demonstrated a lack of PE-PGRS secretion in the presence of an intact *ppe38-71* operon and ESX-5 secretion system (14, 15). While it is clear that the *ppe38-ppe71* operon is involved in PE-PGRS secretion, this phenotype can be present in other mycobacteria, driven by an independent and currently unknown mechanism. By utilising a *ppe38-*71 mutant in *M. tuberculosis*, the collective role of the PE-PGRS and PPE-MPTR proteins that are dependent on both ESX-5 and PPE38 can be studied in the context of host-pathogen interactions. Interestingly, a hypervirulent phenotype was observed with increased bacillary growth of an *M. tuberculosis ppe38-71* mutant in BALB/c mice (13). A similar phenotype was observed in zebrafish infected with a *ppe38* transposon mutant of *M. marinum* (13, 16). It was further demonstrated that an *M. marinum ppe38* transposon mutant is able to modulate the innate immune response in murine macrophages and to alter antigen presentation (17). However, no differences were observed in TNF-α and CD40 levels in murine bone marrow-derived dendritic cells infected with an *M. tuberculosis Δppe38-71* mutant compared to wild type (18). The dispensability of *ppe38*, and by association the secretion of PE-PGRS proteins, is an unexpected observation as these proteins are hypothesised to be important for adaptation to an intracellular lifestyle.

To further explore the role of PE-PGRS and PPE-MPTR proteins in host-pathogen interactions, we characterised the temporal proteome profile of the THP-1 macrophage-like cell line in response to infection with *M. tuberculosis* CDC1551 and an isogenic *ppe38-71* mutant strain. We observed altered pro-inflammatory responses in macrophages infected with *M. tuberculosis Δppe38-71*. We further used stable isotope labelling of amino acids in cell culture (SILAC)-based proteomics to investigate protein turnover rates in response to infection and could show an increased turnover of proteins involved in pro-inflammatory responses in macrophages infected with *M. tuberculosis* CDC1551. Finally, our results suggest a role for PPE38-controlled PE/PPE proteins of *M. tuberculosis* in infection by modulating the inflammatory response through nuclear factor kappa B (NF-kB) signalling *via* the RelB pathway. By combining different approaches, we provide a deeper understanding of the molecular mechanisms exploited by *M. tuberculosis* to alter protective host responses during infection of macrophages.

## Materials and Methods

### Bacterial Strains

*Mycobacterium tuberculosis* CDC1551 *Δppe38-*(further referred to as *Δppe38-71*) and *M. tuberculosis* CDC1551 *Δppe38-71*::pMV*_ppe38-71* (further referred to as complemented) were generated from *M. tuberculosis* CDC1551 as parental strain with an integrated *ppe38-ppe71* serving as the complement under the constitutively expressed *hsp60* promotor. All three strains were obtained from a previous study (13). All bacterial strains were grown in modified Sauton’s medium (0.4% L-asparagine, 0.4% glucose, 0.2% citric acid, 0.05% monopotassium phosphate, 0.05% magnesium sulphate, 0.005% ferric ammonium citrate and 0.001% zinc sulphate, pH 7.0), supplemented with 0.05% Tween-80 at 37°C without shaking in 75 cm^2^ tissue culture flasks. *M. tuberculosis Δppe38-71* was cultured in the presence of 50 µg/ml hygromycin (ThermoFisher, MA, USA) and *M. tuberculosis Δppe38-71:*:pM*V_ppe38-71* was cultured in the presence of 25 µg/ml kanamycin (Sigma-Aldrich, MO, USA) and 50 µg/ml hygromycin as indicated. Antibiotics were only used during pre-culture and were omitted during sub-culturing. *In vitro* growth was monitored by culturing *M. tuberculosis* CDC1551, *Δppe38-71* and the complemented strain as stated above and measuring optical density at 600 nm over 20 days.

**Figure 1:**
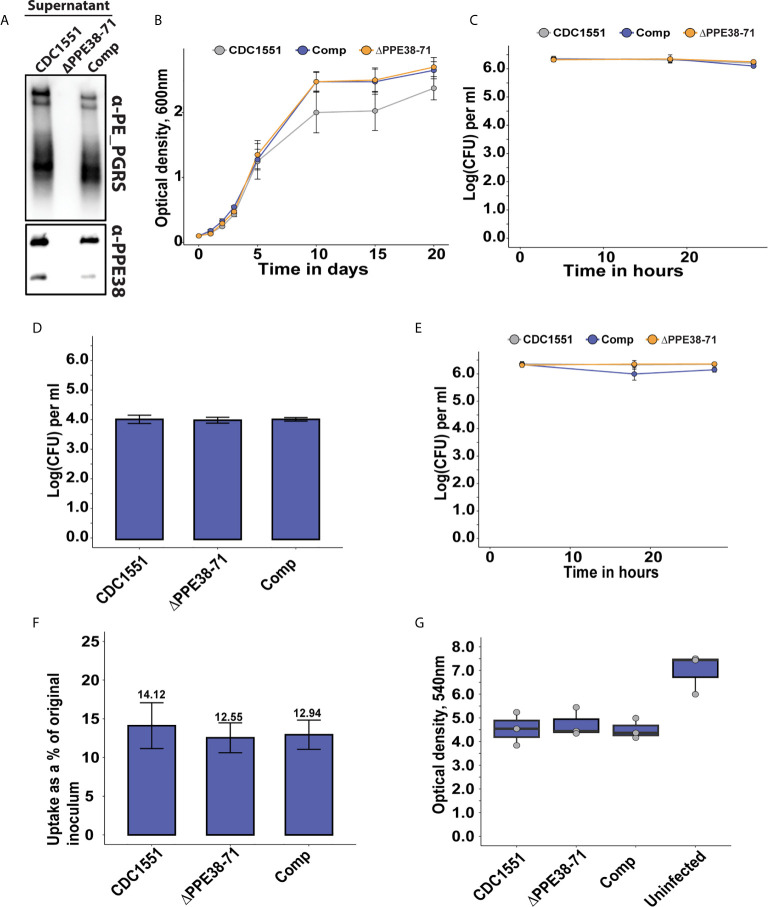
The absence of PPE38 controlled PE-PGRS proteins has no effect on bacterial survival or macrophage death in a 28 hour period. **(A)** PE-PGRS and PPE38 immunoblot of *in vitro* culture supernatants of *M. tuberculosis* CDC1551, *Δppe38-71* and the complemented strain harvested at OD_600_ of ~1. **(B)** *In vitro* growth curves of *M. tuberculosis* CDC1551, *Δppe38-71* and the complemented strain by optical density measurements for 20 days. Data is representative of four independent experiments and error bars represent SEM. Two-way ANOVA with Tukey HSD post-hoc testing was used to determine a significant difference with a q-value cut-off at 0.05 **(C)** Intracellular growth curve of *M. tuberculosis* CDC1551, *Δppe38-71* and the complemented strain in THP-1 macrophage-like cells. Growth was monitored over 28 hours and infected macrophages were harvested at 4 hours, 18 hours and 28 hours after exposure to mycobacteria. Mycobacterial load was measured by CFU enumeration. Data is representative of three independent experiments and error bars represent SEM. **(D)** Extracellular bacteria at 3 hours post-infection were determined by CFU enumeration after 7 consecutive washes. No significant difference was detected by one-way ANOVA between the different strains. Data is representative of three independent experiments and error bars are indicative of SEM. **(E)** Differential uptake of mycobacteria by THP-1 macrophage-like cells during infection was measured by washing excess bacilli at 3 hours post-infection. Pen/Strep treatment for 1 hour was used directly before each harvesting time point, i.e. 4 hours; 18 hours and 28 hours; to remove any extracellular bacteria still present. The intracellular mycobacterial load was determined by CFU enumeration and data is representative of three independent experiments, error bars represent SEM. **(F)** Percent bacterial uptake calculated from the original inoculum at an MOI of 3:1. Data is representative of three independent experiments where the percentage was calculated from the mean CFU of each strain obtained from 4 hours post-infection divided by the mean CFU of the original inoculum. **(G)** THP-1 macrophage viability was measured by MTT assay at 18 hours post-infection. Data is representative of three independent experiments and error bars represent SEM. One-way ANOVA was used to determine significant differences with a p-value cut off of 0.05.

The mean was derived from four (n = 4) biologically independent experiments and error bars represent standard error of the mean (SEM). Statistical differences between strains at each time point was determined by two-way ANOVA followed by a Tukey HSD post-hoc test. The q-value used to infer statistically significant difference was set at 0.05, thus only a q-value below this number was considered significant. The statistical tests were performed using the R statistical programming language version 3.8.1 using the standard library. See **Figure 1B** and the corresponding legend for detailed statistical descriptions related to each figure.

### Cell Lines

The THP-1 human monocytic cell line (ATCC^®^TIB-202^©^) available from the American Type Culture Collection (ATCC, VA, USA) was stored as frozen seed lots at -80°C suspended in cell freezing media (Merck, NJ, USA) until use. The THP-1 cells were cultured in RPMI (ThermoFisher, MA, USA) media supplemented with 10% foetal bovine serum (FBS) (ThermoFisher, MA, USA), further referred to as R10 media, at 37°C and in a 5% CO_2_ atmosphere for a maximum of three passages in either 25 or 75 cm^2^ tissue culture flasks. For pulse-chase SILAC (pSILAC) experiments, the THP-1 monocytes were grown as described above, however, the 10% FBS was substituted for 10% dialysed FBS (R10_LIGHT_). THP-1 monocytes were differentiated into macrophage-like cells using 50 ng/ml phorbol myristate acetate (PMA) (Sigma-Aldrich, MO, USA) for three days. For experiments in *ex vivo* bacterial growth, 48-well tissue culture grade plates were used with 1 x 10^5^ THP-1 seeding density. Proteomics experiments involving THP-1 cells used six well plates with a seeding density of 1 x 10^6^ cells The media was changed after three days, the cells washed twice with phosphate-buffered saline (PBS) pH 7.4 (ThermoFisher, MA, USA) and the media replaced with either R10 or R10_LIGHT_. The THP-1 macrophage-like cells were subsequently rested for 24 hours at 37°C in a 5% CO_2_ atmosphere before performing further experiments.

### Macrophage Infection

*M. tuberculosis* CDC1551, *Δppe38-71* and the complemented strain were grown, separately, as described above for 4 days or when an OD_600_ of 1.0 was reached. The cells were harvested by centrifugation (4000 rpm, 10 min) and washed three times with PBS pH 7.4 to remove Tween-80. Mycobacterial cells were subsequently resuspended in 5 ml PBS pH 7.4 and sonicated for 10 minutes in a water bath sonicator, followed by filtering through a 40 µm cell strainer to remove bacterial clumps. Optical density (OD) was measured, and suspensions were diluted to an OD_600_ of 0.1 (corresponding to approximately 1 x 10^7^ cells/ml) in R10 medium.

The THP-1 macrophage-like cells were infected with the different *M. tuberculosis* strains at a multiplicity of infection (MOI) of 3:1 and incubated for 3 hours at 37°C in a 5% CO_2_ atmosphere to allow for uptake of mycobacteria. For label-free proteomics, the infected macrophages were washed seven times with PBS pH 7.4 to remove extracellular bacteria and proteins were harvested at 4 hours and 18 hours post-infection, as described below. For colony forming unit (CFU) determination, the infected macrophages were treated with 100 U/ml pen-strep (penicillin/streptomycin) at 37°C for 1 hour, followed by three washes with PBS pH 7.4. Macrophages were lysed using deionised water at the indicated time points. The lysates were serially diluted to a maximum dilution of 1 x 10^-6^ and plated on 7H11 agar, followed by incubation at 37°C for ~3 weeks or until colonies formed. To account for the presence of extracellular bacteria, THP-1 macrophage-like cells were washed seven times with PBS pH 7.4 to remove mycobacteria that were not internalised after three hours of infection. Bacteria remaining after the consecutive washes were determined by plating the final wash on 7H11 agar and incubated at 37°C for ~3 weeks or until colonies formed.

The bacterial viability and growth within macrophages were determined from three independent experiments described in **Figures 1C–E** and the corresponding legend. Data was gathered by infecting THP-1 macrophage-like cells with mycobacterial strains and enumerated by colony forming unit (CFU) counts. The results were derived from three biologically independent experiments (n = 3) and error bars represent SEM. Hypothesis testing was performed in the R statistical programming language version 3.8.1 using two-way ANOVA with a Tukey HSD *post hoc* test for data represented in **Supplementary Figures S1C, E** while a one-way ANOVA with a Tukey HSD *post hoc* test was used for **Figure 1D**. Statistical significance was inferred using the resulting q-value and a cut-off was set at 0.05.

For pSILAC experiments, THP-1 macrophage-like cells were washed three times with PBS pH 7.4 and the media replaced with SILAC RPMI devoid of lysine and arginine (ThermoFisher, MA, USA). The cells were subsequently incubated for 3 hours at 37°C in a 5% CO_2_ atmosphere prior to infection to facilitate amino acid uptake upon exposure to mycobacteria. After the three-hour incubation, the SILAC RPMI was replaced with fresh SILAC RPMI supplemented with 10% dialysed FBS (ThermoFisher, MA, USA), 0.4 mg/ml N415C613-arginine and 0.08 mg/ml N215C613-lysine (R10_HEAVY_) and placed on ice until infection.

*M. tuberculosis* CDC1551, *Δppe38-71* and the complemented strain were cultured and prepared for infection as previously described, with minor modifications. Briefly, the mycobacterial cells were harvested, sonicated and filtered as described above, however, the cells were diluted to an OD_600_ of 0.1 in R10_HEAVY_ medium. Macrophage infections were further carried out as described above. Proteins were harvested at 4 hours, 8 hours, 12 hours, 18 hours and 28 hours post-infection for protein turnover analysis, as described below.

### MTT Assay

The 3-(4,5-dimethylthiazol-2-yl)-2,5-diphenyltetrazolium bromide (MTT) reagent was prepared in R10_Light_ to a final concentration of 5 mg/ml. Supernatants of infected as well as uninfected macrophages were removed following infection at 18 hours and replaced with R10_Light_-MTT media. The macrophages were subsequently incubated at 37°C, 5% CO_2_ atmosphere for two hours. The media containing MTT was removed after this incubation step and replaced with DMSO and incubated at 37°C, 5% CO_2_ atmosphere for an additional 15 minutes. Macrophage viability was calculated by colorimetric shift measured at 540 nm using a plate reader.

The viability assay is described in **Figure 1G**, the corresponding legend and details of the statistical test can be found in **Data Sheet 1: Table S1**. Results were gathered by infecting THP-1 macrophage-like cells with each mycobacterial strain as well as an uninfected control and measuring optical density at 18 hours post infection. The data in this experiment was derived from three (n = 3) independent experiments and significant differences were detected by one-way ANOVA following a Tukey HSD *post hoc* test. This test was performed in the R statistical programming language version 3.8.1 using the standard library and a q-value less than 0.05 was considered significant.

### Protein Extraction and Proteomic Sample Preparation

At each time point, infected THP-1 macrophage-like cells were washed four times with PBS pH 7.4 on ice. Modified RIPA buffer (50 mM Tris-HCl pH 7.4, 150 mM NaCl, 1mM EDTA, 1% IGEPAL CA-630, 0.1% sodium deoxycholate, Benzonase and Roche Complete EDTA-free protease inhibitor) was added to each well and placed on ice for 10 minutes to lyse cells. The lysates were subsequently centrifuged (14 000 rpm, 20 min) to remove cellular debris. Proteins were precipitated by the addition of four volumes ice-cold acetone and quantified by modified Bradford assay (19).

All bacterial strains were grown in modified Sauton’s medium (0.4% L-asparagine, 0.4% glucose, 0.2% citric acid, 0.05% monopotassium phosphate, 0.05% magnesium sulphate, 0.005% ferric ammonium citrate and 0.001% zinc sulphate, pH 7.0), supplemented with 0.05% Tween-80 at 37°C without shaking. For tween free supernatants, the bacteria was grown in tween until an OD of ~1 was reached and the detergent was removed by sequential washing through centrifugation (4000 rpm, 20 minutes) for a total of 4 washes and sub-cultured in Sauton’s media without Tween-80. The bacteria was cultivated for an additional 5 days at 37°C before harvesting. Bacterial whole-cell lysates were produced by separating the cells from the supernatant by centrifugation (4000 rpm, 4°C, 10 minutes). The supernatant was filter sterilised using 0.22 µm low bind syringe filters (Merck Milipore, NJ, USA) and used for the Tween-80 supernatant fraction. The pellet was resuspended in 1 ml ice cold protein extraction buffer (10 mM Tris-HCl pH 7, 0.1% Tween-80, Complete Protease inhibitor cocktail table) and centrifuged once more (14 000 rpm, 4°C, 2 minutes). The pellet was suspended in 300 µl cold protein extraction buffer supplemented with RNase-free DNaseI and transferred to cryogenic vials containing an equal volume 0.1 mm acid washed glass beads. The suspension was mechanically lysed by bead-beating for 30 second intervals with 30 seconds on ice for a total of 8 cycles. The resulting lysate was clarified by centrifugation (14 000 rpm, 4°C, 15 minutes) and filter sterilised through 0.22 µm pore acrodisks and syringes. Tween-free supernatants were harvested by separating the cells from the supernatant using 40 µm cell strainers (Corning, NY, USA) followed by centrifugation (4000 rpm, 10 minutes) and filter sterilised using 0.22 µm Steriflip^®^ filters (Sigma-Aldrich, MI, USA). The supernatants were concentrated using Amicon Ultra 3 KDa spin columns and precipitated with ice cold acetone overnight. The precipitated proteins were resuspended in 8M urea, and all proteins were quantified using a modified Bradford assay and used for LC-MS/MS (19).

Equal amounts of protein (20 µg) were used for sample preparation for LC-MS/MS following a tube gel protocol (20). Briefly, proteins suspended in 8M urea were diluted in 1M tris-HCl pH 6.8 and cast in an acrylamide gel containing 10% SDS within an Eppendorf tube. The samples were allowed to set overnight at room temperature. Protein-containing gels were removed from the Eppendorf tubes and cut into ~1 mm x 1 mm gel pieces. Gel pieces were washed three times with 50 mM ammonium bicarbonate (Sigma-Aldrich), followed by reduction with 5 mM tris(2-carboxyethyl)phosphine (Sigma-Aldrich, MO, USA) for 1 hour at 45°C. After reduction, the proteins were alkylated with 55 mM iodoacetamide (Sigma-Aldrich, MO, USA) for 1 hour at room temperature. The gel pieces were washed twice with 100% acetonitrile after alkylation. Sequencing grade modified trypsin (Promega, WI, USA) was added to the gel pieces at a 1:50 trypsin to protein ratio and incubated at 4°C for 1 hour, followed by an 18 hours proteolytic digestion at 37°C in a humidified chamber. Peptides were eluted by sequential addition of 50%, 70% and 100% acetonitrile until gel pieces turned opaque. The eluates were then dried in a vacuum desiccator (SpeedVac). Dried peptides were suspended in 5% acetonitrile (Sigma-Aldrich, MO, USA) containing 0.1% formic acid and desalted using C18 desalting columns (ThermoFisher, MA, USA) as recommended by the manufacturer.

### LC-MS/MS

Dried peptides were dissolved in 30 µl of solvent A (2% acetonitrile containing 0.1% formic acid in HPLC-grade water) and 500 ng peptides was analysed. All chromatography was performed on a nanoAcquity UPLC system (Waters, MA, USA) using a 200 cm uPAK™ column (Pharmafluidics, Ghent, Belgium) coupled to a Thermo Q-Exactive Plus Orbitrap mass spectrometer (ThermoFisher, MA, USA) equipped with a Flex nanoelectrospray source. The spray voltage was set to 1.9 kV (Thermo Fisher, MA, USA) and the capillary temperature was 250°C. Peptide separation was performed using a linear gradient of solvent B (98% acetonitrile, 0.1% formic acid and 2% HPLC grade water), starting with 3% solvent B and increased to 40% solvent B over 80 minutes. Solvent B was increased to 100% in 5 minutes and subsequently decreased to 3% solvent B in 5 minutes. Solvent B was kept at 3% B for an additional 35 minutes at a flow rate of 750 nL/min, where a column temperature of 50°C was maintained with a heater. Mass spectrometry was performed in data-dependent acquisition mode using a full MS1 scan (350-1850 m/z, resolution at 70000, max injection time was 100 ms and ACG target was 3e6), and selecting precursor ions with a 2^+^ or greater charge state for MS/MS analysis. This was followed by HCD fragmentation with normalised collision energy set at 28% and MS/MS acquisition (200–2000 m/z, resolution 17,500, max injection time of 80 ms, AGC target was 1e5) of the top 20 most intense precursors from each full scan. Dynamic exclusion of ions was implemented using a 20s exclusion duration and only ions with an unassigned charge state were disregarded.

### Mass Spectrometry Data Analysis

**Figure 2:**
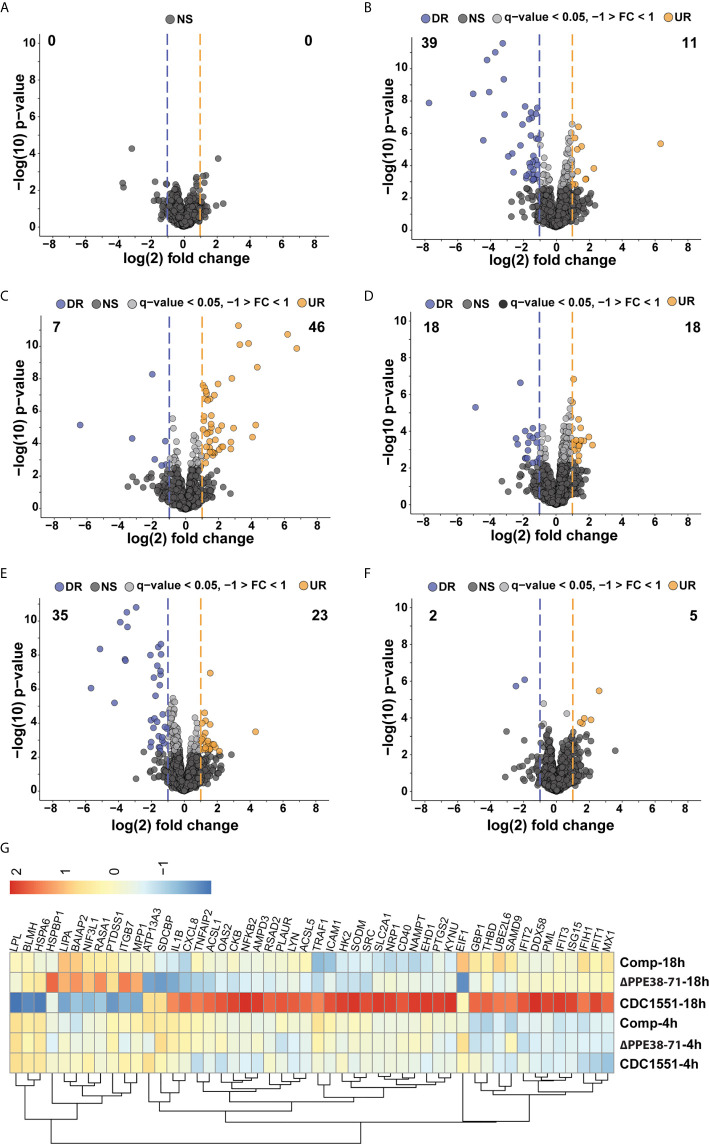
Label-free proteomic analysis reveals altered inflammatory responses in *M. tuberculosis*-infected macrophages. Volcano plots representing differential protein abundance in macrophages infected with *M. tuberculosis* CDC1551 compared to infection with *Δppe38-71* at **(A)** 4 hours post-infection, **(B)** 18 hours post-infection, **(C)** infection with *M. tuberculosis* CDC1551 at 18 hours compared to 4 hours and **(D)** infection with *Δppe38-71* at 18 hours compared to 4 hours post infections. Significance cut-offs were set at a q-value less than 0.05 and a log2 fold change greater than 1. Numbers indicate the amount of significantly regulated proteins. **(E)** Volcano plot representing regulated proteins of THP-1 macrophage-like cells infected with the complemented strain compared to *M. tuberculosis* CDC1551 at 18 hours post-infection. Significance cut-offs were set at a q-value less than 0.05 and a log2 fold change greater than 1. **(F)** Volcano plot representing proteins of THP-1 macrophage-like cells infected with the complemented strain at 18 hours compared to 4 hours post infection. Significance cut-offs were set at a q-value less than 0.05 and a log2 fold change greater than 1. **(G)** Heat map of the main regulated proteins between the groups, displaying the log_2_ fold changes between THP-1 macrophage-like cells infected with *M. tuberculosis* CDC1551, *Δppe38-71* and the complemented strain at 18 hours post-infection. LFQ intensities were Z-scored and are from three independent biological replicates.

All tandem mass spectra were analysed using MaxQuant version 1.6.10 (21) and searched against the human proteome (UP000005640, containing 74 349 entries) database downloaded on 14/10/2017 from Uniprot and the *M. tuberculosis* CDC1551/Oshkosh proteome (UP000001020, containing 4 204 entries) downloaded on 17/4/2017. Peak list generation of label-free tandem mass spectra was performed within MaxQuant using default parameters and the built-in Andromeda search engine (22). Enzyme specificity was set to consider fully tryptic peptides with two missed cleavages were allowed. Oxidation of methionine and N-terminal acetylation were allowed as variable modifications. Carbamidomethylation of cysteine was allowed as a fixed modification. A protein and peptide false discovery rate of less than 1% was employed in MaxQuant with match between runs enabled. Proteins that contained similar peptides that could not be differentiated on the basis of MS/MS analysis alone were grouped to satisfy the principles of parsimony. Data handling, statistical tests and figure generation was performed using ProVision, an online data analysis platform that uses the LIMMA package in R for statistical tests (23). Briefly, reverse database hits, contaminants and proteins only identified by site modifications were removed. Precursor intensity values for each protein was obtained from MaxQuant using the MaxLFQ algorithm available internally (24). The file was further filtered for each protein group to contain at least two unique peptides. The assigned LFQ intensity values were subsequently log2 transformed to gain a normal distribution and further filtered for two values in at least one group. This resulted in the high confidence expression dataset, and missing values were imputed from a truncated normal distribution of transformed LFQ intensities. Quantile of quantile plots were used within the ProVision application to check for normality prior to statistical testing. Multiple hypothesis testing was corrected using the Benjamini-Hochberg FDR set at 0.05, and a two-fold cut-off was implemented. The statistical analysis and visualisations of label free mass spectrometry data can be found in **Supplementary Figure S1** and **Figure 2** with an extended analysis in **Supplementary Figure S2**. The mass spectrometry data pertaining to this experiment, which was used to generate these figures are available in **Data Sheet 1** and **Table S2**. Differential expression of *in vitro* grown mycobacterial strains was as described in **Figures 5E, F** and **Supplementary Figures S5A, B** as well as **Tables S5–7**. The data used for hypothesis testing was derived from three (n = 3) biologically independent experiments for each condition. Significant differences were accepted when the q-value was below 0.05 and a log2 fold change of 1.

### Protein Turnover

The search parameters for SILAC were largely similar to that of the label-free searches using MaxQuant version 1.6.10 and the same human proteome (UP000005640) as a reference database. For the pSILAC searches a multiplicity of 2 was chosen adding heavy arginine with a mass shift of 10 Da and heavy lysine with a mass shift of 8 Da. Furthermore, the re-quantify function was disabled and match between runs was enabled. Oxidation of methionine and N-terminal acetylation was chosen as variable modification and carbidomethylation of cysteine was set as the fixed modification and all FDR cut offs for peptide identification was set at 0.01. Under conditions with increased arginine cell lines can convert the excess arginine to proline which results in non-specific dilution in the stable isotope signal and can confound intensity values. We therefore tested this conversion in our differentiated THP-1 macrophage-like cell line with LPS stimulating with heavy lysine and arginine for 18 hours. Searching in the same manner as above with proline (6 Da shift) as a variable modification only identified 5 proteins containing this amino acid. Thus, we continued with the 0.4 mg/ml N415C613-arginine for the rest of our experiments. The resulting protein groups file was filtered in the same manner as detailed above for the label-free proteomics. Briefly, all contaminants; reverse database hits as well as proteins only identified by site modifications was removed from the SILAC dataset and we additionally filtered for a minimum of two unique peptides. The post-filtered raw ratios of each strain and each time point was used for further analysis.

As the heavy isotopes increase, the light isotopes decay providing a function to extrapolate half-life for the population of proteins based on linear regression. Protein turnover was calculated from an average from the raw ratios using a similar approach as previously described (25). As the natural occurrence of N215C613-lysine is exceedingly rare we assumed a zero time point where the heavy fraction was set to zero for further handling in a manner as previously described (26). The raw ratios of each condition (i.e. THP-1 infected with CDC1551, *Δppe38-71* etc.) were filtered to contain at least three ratios in the time course and with a coefficient of determination above 0.85. The half-lives (T_1/2_) of each protein were determined using a first order reaction equation (eq 1). This was subsequently derived for calculating the half-life (eq 2), where half-life is equal to the natural log of two divided by the rate (K_dp_).

(1) $$N_{t}=N_{0}e^{-K_{dp}t}$$

(2) $$T_{\frac{1}{2}}=\frac{ln(2)}{K_{dp}}$$

The reaction rate (K_dp_) was calculated using equation 3 without accounting for dilution by replication as THP-1 macrophage-like cells are terminally differentiated. Here *r* is representing the raw ratio at each time point and *t_i_* is each specific time point. For optimal handling the equation can be expressed using all variables for a direct calculation of half-life (eq 4).

(3) $$K_{dp}=\frac{\Sigma \;ln(r+1)t_{i}}{\Sigma t_{i}^{2}}$$

(4) $$T_{\frac{1}{2}}=ln(2)÷\left(\frac{\Sigma \;ln(r+1)t_{i}}{\Sigma t_{i}^{2}}\right)$$

Calculating the Kdp values were implemented using the Excel macro LinEstGap which implements equation 3 (see data availability for link to the macro) and the half-lives were calculated using equation 4. The average half-life was calculated after filtering for each replicate and was further processed in the R statistical programming language. Here the average half-life was calculated per condition which produced a log normal distribution and missing values. This was followed by filtering the dataset where a protein should contain at least one half-life representing at least one of the four conditions.

**Figure 3:**
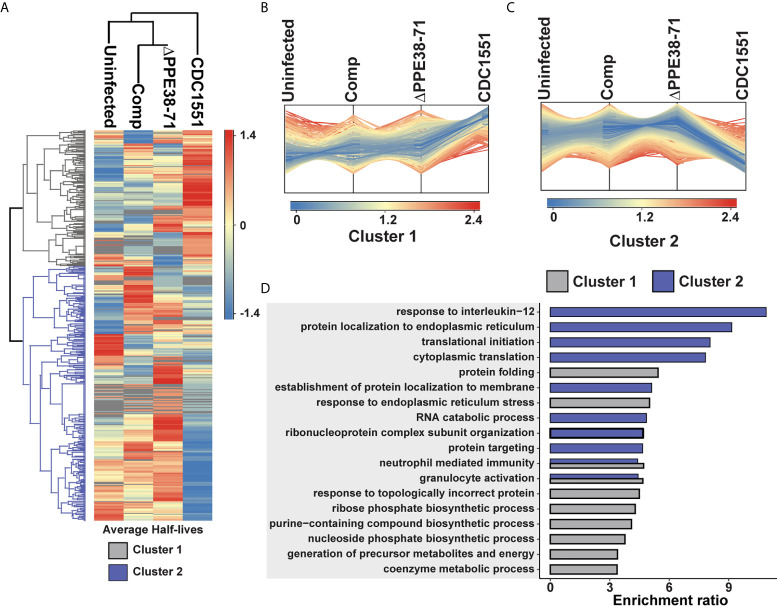
Infection with *M. tuberculosis* CDC1551 and not *M tuberculosis Δppe38-71* causes a shift in protein turnover associated with the IL-12 pro-inflammatory pathway. **(A)** Half-lives from THP-1 macrophage-like cells infected with *M. tuberculosis* CDC1551, *M. tuberculosis Δppe38-71* and the complemented strain and uninfected control macrophages were assessed using hierarchical clustering with Euclidean distance. Two distinct clusters formed representing either an increase (cluster 1) or a decrease (cluster 2) in protein half-lives as depicted by profile plots. Protein groups from **(B)** cluster 1 and **(C)** cluster 2 are represented as a deviation in the estimated marginal mean of each protein half-life, after adjustment of covariates, across each factor. **(D)** GO enrichment analysis of each cluster obtained in A and B. Protein turnover data is representative of two biological replicates.

**Figure 4:**
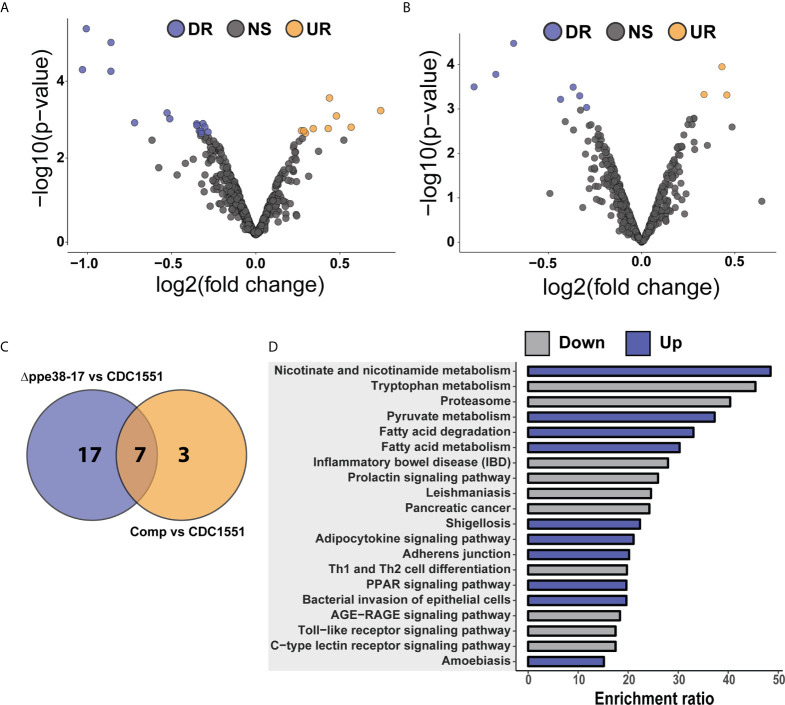
Differential regulation of individual protein half-lives point towards changes in the inflammatory response. Volcano plot depicting altered protein half-lives between **(A)** *M. tuberculosis* CDC1551- and *Δppe38-71-*infected macrophages or **(B)** *M. tuberculosis* CDC1551- and complement-infected macrophages. Significantly altered protein half-lives are highlighted based on q-value set at 0.05. Downregulated proteins are denoted as DR (purple), upregulated as UR (orange) and non-significant as NS (grey) in the volcano plots. **(C)** Venn diagram depicting the number of macrophage protein half-lives present either uniquely or commonly between the *M. tuberculosis* CDC1551 infected and *Δppe38-71* infected macrophage and between *M. tuberculosis* CDC1551 infected and complement-infected macrophage comparisons. Data is representative of two biological replicates. **(D)** Over-representation analysis of the proteins with differentially regulated half-lives against KEGG pathways, where up is indicative of rapid turnover and down indicative of slower turnover. Over-representation was calculated using the WebGestalt API using default parameters.

For further analysis we Z-scored the half-lives to obtain a dataset used to generate heatmaps where the row clustering was to gain a distance from the centroid metrics. These clusters were enriched for gene KEGG pathways using the WebgestaltR package available on the CRAN repository as shown in **Figure 3D** (27). Hypothesis testing was used to determine differentially regulated half-lives from two biologically independent experiments (n = 2) across five time points namely 4 hours, 8 hours, 12 hours, 18 hours and 28 hours post infection. The difference in protein turnover between CDC1551 and *Δppe38-71* infected THP-1 macrophage-like cells was determined using hypothesis testing. This allowed for stringent filtering, i.e. contains ratios in both replicates in both conditions, without losing valuable data. The strict filtered dataset for each comparison was log2 transformed to obtain a normal distribution and the LIMMAf package was used for hypothesis testing. The resulting p-values were corrected for using Benjamini-Hochberg FDR set at 0.05 and fold change was disregarded for these specific analyses. The results of these tests were visualised in **Figures 4A, B**, the raw data used to generate these figures are available in **Data Sheet 1** and **Table S4**. The differentially regulated proteins were further used to enrich for KEGG pathways using the WebGestaltR package as shown in **Figure 4D**.

**Figure 5:**
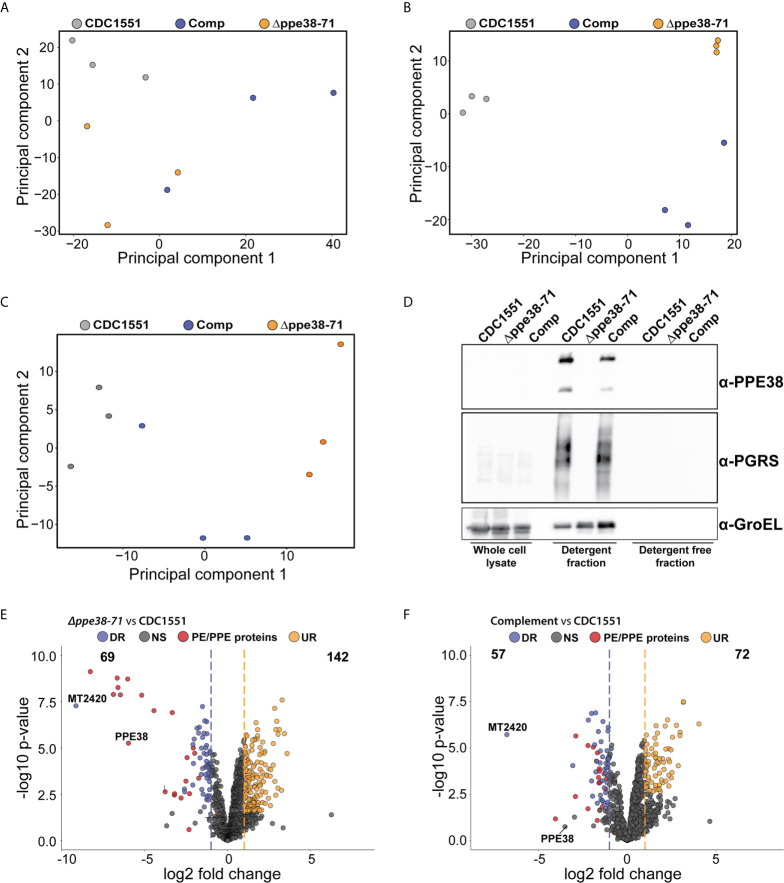
*In vitro* proteomics of *M. tuberculosis* strains show partial complementation that can be restored by the omission of detergent. Principal component analysis of **(A)** whole-cell lysates, **(B)** supernatant containing detergent and **(C)** supernatant without detergent of each *M. tuberculosis* strain after four days of cultivation. **(D)** Western blot of each fraction shows the PE-PGRS proteins in the supernatant only when there is detergent present in the growth media. Volcano plots of supernatants containing detergent from **(E)** *M. tuberculosis Δppe38-71* and **(F)** the complemented strain compared to *M. tuberculosis* CDC1551 when cultivated in detergent. The q-value was set to 0.05 and the log2 fold change was set to 1. The data is representative of three independent experiments.

### Western Blots

Macrophage whole-cell lysates and bacterial supernatants were either probed with a WesternBreeze anti-rabbit chemiluminescent kit (Thermo Fisher, MA, USA) or manually if primary antibodies required an anti-mouse secondary antibody. Briefly, whole-cell lysates were quantified using a modified Bradford assay and 50 µg total protein content was separated by SDS-PAGE. Proteins were subsequently transferred to nitrocellulose membranes for 1 hour. The membranes were stained with Ponceau S (0.1%, w/v in 5% acetic acid) to inspect sample loading. Membranes were probed with anti-GAPDH (CST, D16H11), anti-IL-1B (CST; D3U3E), anti-NF-kB1 (CST; D4P4D) anti-NF-kB2 (CST,18D10), anti-ISG15 (CST; 22D2), anti-DDX58 (CST; D14G6), anti-PPE38 (custom, Innovagen, Sweden) or anti-PGRS (3) overnight at 4°C, as indicated in the text. This was followed by incubation with an alkaline phosphatase-conjugated secondary anti-rabbit antibody or a horseradish peroxidase-conjugated goat anti-mouse secondary antibody for 1 hour at room temperature. Blots were visualised using the chemiluminescent substrate provided by the WesternBreeze kit or with ECL detection reagent (Bio-Rad) and imaged on a ChemiDoc Imaging System (Bio-Rad).

### Macrophage Stimulations

*M. tuberculosis* CDC1551, *Δppe38-71* and complemented strains were grown in Sauton’s media containing 0.05% Tween-80 for 5 days or until an OD_600_ of ~1 was reached. *M. tuberculosis* culture supernatants were harvested by centrifugation (4000 rpm, 10 min) and concentrated using 4 kDa molecular weight cut off Amicon Ultra spin columns (Merck Millipore, MA, USA). Proteins in the cell-free supernatants were precipitated using ice-cold acetone and kept overnight at -20°C. The precipitated proteins were harvested by centrifugation (14000 rpm, 30 min) and resuspended in 8M urea in 50 mM triethylammonium bicarbonate (urea buffer). Protein concentration was determined using a modified Bradford assay, as described previously (19).

THP-1 monocytes were seeded into 6-well plates at 1 x 10^6^ cells/well and differentiated into macrophages, as described above. THP-1 macrophage-like cells were stimulated for 18 hours with lipopolysaccharide from *E. coli* (Sigma-Aldrich, MO, USA) at 100 ng/ml or proteins from cell-free supernatants of *M. tuberculosis* at 20 µg per well. Unstimulated cells served as baseline controls for IL-1B expression. Whole-cell lysates of the macrophages were harvested as described above and stored at -80°C until further use.

Expression was quantified by densitometry of western blots, where a represented blot is depicted in **Supplementary Figure S5C**. Relative quantification was determined from three independent biological experiments (n = 3) and depicted in **Supplementary Figure S5D**. Significant differences were determined by one-way ANOVA followed by Tukey HSD and details are depicted in **Supplementary Figure S5E**. Significant differences were accepted when the q-value was below 0.05.

### Immunofluorescent Staining and Microscopy

THP-1 monocytes were cultivated, differentiated and infected as described above. After 18 hours of infection, the medium was removed, the cells were fixed in 4% paraformaldehyde (PFA) for 30 minutes at room temperature and washed three times in PBS pH 7.4 for 10 minutes. Cells were permeabilised using 0.1% Triton X-100 in PBS pH 7.4 for 10 minutes at room temperature and washed three times in PBS pH 7.4 for 10 minutes. Cells were blocked with 1% bovine serum albumin (BSA) in PBS pH 7.4 containing 0.1% Tween-80 and 0.1M glycine for 1 hour at room temperature, after which cells were incubated with corresponding primary antibodies diluted in 1% BSA in PBS pH 7.4 overnight at 4°C in a humidified chamber. Antibody dilutions were used as follows: RelA antibody (NF-kb p65 (D14E12) XP^®^ Rabbit mAb, Cell Signalling Technology, MA, USA) was used at a 1:400 dilution; RelB (Recombinant Anti-Rel B antibody [EPR7076] - C-terminal (ab180127, Abcam, Cambridge, UK) was used at a 1:200 dilution; NF-kb1 p105/p50 (D4P4D, Rabbit mAb, Cell Signalling Technology, MA, USA) was used at a 1:200 dilution; NF-kb2 p100/p52 (D7AK9, Rabbit mAb, Cell Signalling Technology, MA, USA) was used at a 1:400 dilution. Cells were washed three times in PBS pH 7.4 for 10 minutes and incubated with anti-rabbit Alexa Fluor 488 secondary antibody (Thermo Fisher, MA, USA) used at 1:500 dilution for 1 hour at room temperature in the dark. Cells were washed three times in PBS pH 7.4 for 10 minutes and subsequently stained with Phalloidin–Tetramethylrhodamine B isothiocyanate (Sigma Aldrich, MI, USA) to visualize F-actin. Nuclei were counterstained with Hoechst in PBS pH 7.4 for 10 minutes at room temperature in the dark. Cells were washed a further three times in PBS pH 7.4 for 10 minutes before mounting upside down onto glass microscope slides using Dako fluorescent mounting medium and air drying overnight in the dark at room temperature. Slides were stored at 4°C in the dark until imaging. Unstained, single stained and secondary antibody only controls were prepared for each experiment to assess background autofluorescence and signal specificity in each channel. Images were obtained using a Carl Zeiss LSM 780 confocal microscope (Plan-Apochromat x63/1.40 oil DIC M27 objective lens). Images were acquired using the ZEN software (Carl Zeiss, Oberkochen, Germany). Acquisition settings for imaging were identically set for all treatment groups within each experiment. Analysis and quantification of images were done with the ZEN black software version 13.0.0.518 (Carl Zeiss, Oberkochen, Germany) and any manipulations done, such as min/max, were extended to all channels. No single channel enhancements were used during quantification and all changes were applied equally across the entire image. In addition, twenty cells were chosen from at least 10 random fields per replicate to with a minimum of three independent experiments (n=3) to generate the results as depicted in **Figure 7I**. For display images, channels were enhanced as appropriate to accurately demonstrate nuclear translocation of proteins.

**Figure 6:**
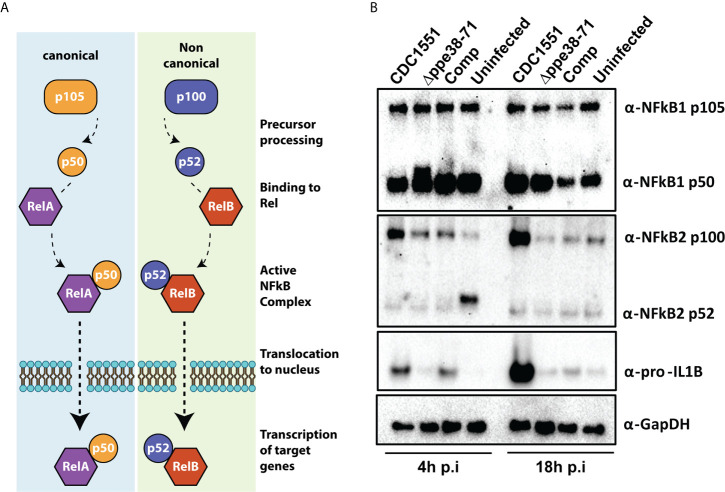
THP-1 macrophage-like cells signal through the canonical NF-kB pathway when infected with *M. tuberculosis* CDC1551. **(A)** Schematic representation of the canonical and non-canonical NF-kB pathways. **(B)** Western blots for components of the NF-kB pathway (α-NF-kB1 and α-NFkB2) and downstream effectors (α-pro-IL1β) as well as their respective sub-units. The western blots were done on protein samples harvested at 4 hours and 18 hours post-infection. α-GapDH was used as a loading control.

**Figure 7:**
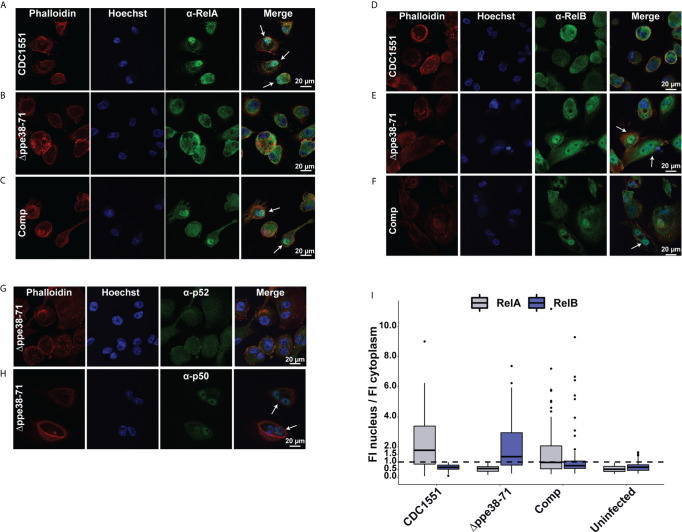
Infection with *M. tuberculosis Δppe38-71* stimulates a RelB/p50 pathway in THP-1 macrophage-like cells. Confocal microscopy images showing individual fluorescent channels probing for F-actin (phalloidin), the nucleus (Hoechst) and **(A–C)** RelA (Alexa Fluor 488) for **(A)** *M. tuberculosis* CDC1551, **(B)** *Δppe38-71*, **(C)** complement-infected macrophages. Macrophages infected with **(D)** *M. tuberculosis* CDC1551, **(E)** *Δppe38-71* and **(F)** the complemented strain were also probed for translocation of RelB (Alexafluor 488). **(G)** Confocal microscopy images showing p100/p52 and **(H)** p105/p50 nuclear translation in *Δppe38-71* infected macrophages. White arrows indicate translocation events. **(I)** Quantification of fluorescence intensity in the nucleus compared to that in the cytoplasm. Twenty cells were chosen from at least 10 random fields per replicate with a minimum of three independent experiments. The dashed line indicates a ratio of 1 for the cut off where translocation occurs.

### ELISA

Supernatants harvested from infected THP-1 macrophage-like cells at 18 hours and 48 hours post-infection were sterilised using a 0.22 µm syringe filter. Sterilised supernatants were subsequently assayed for interleukin 12 p70 (IL-12p70) and interleukin 3 (IL-13) levels using ELISA kits (Biosource, Invitrogen), as indicated by the manufacturer.

**Figure 8:**
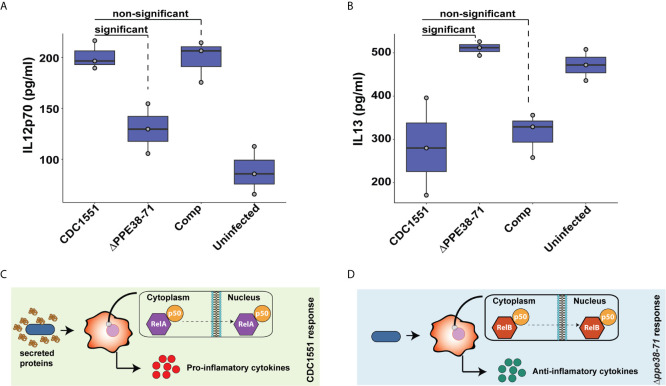
Interleukin-12p70 and IL-13 are differentially secreted by THP-1 macrophage-like cells at 48 hours post-infection. The levels of IL-12p70 **(A)** and IL-13 **(B)** in supernatants of THP-1 macrophage-like cells infected with either *M. tuberculosis* CDC1551, *Δppe38-71* or complemented strains and uninfected macrophages were determined by ELISA. Data is representative of three biological replicates and significant differences were determined with one-way ANOVA as well as a Tukey HSD post-hoc test with a q-value set at 0.05. Schematic illustration of a proposed macrophage response upon infection with **(C)** *M. tuberculosis* CDC1551 and **(D)** *M. tuberculosis Δppe38-71*.

The data was derived from three independent biological experiments (n = 3) and is depicted in **Figures 8A, B** and details of the statistical test can be found in **Figure 8** legend and **Data Sheet I** and**Table S8**. Significant differences were determined by one-way ANOVA followed by Tukey HSD and accepted when the q-value was below 0.05.

## Results

### PPE38 Does Not Influence *M. tuberculosis* Uptake or Replication Within Macrophages

The cell surface of *M. tuberculosis* is covered with pathogen-associated molecular patterns (PAMPs), which will be recognised by the host during infection to initiate immune responses. The PE-PGRS and PPE-MPTR proteins are groups of PAMPs localised on the cell surface of *M. tuberculosis* that have been implicated in host-pathogen interactions and virulence (28–30). Previous work has linked a *Δppe38-71* deletion mutant to increased virulence in *M. tuberculosis* by controlling a subset of PE-PGRS and PPE-MPTR secretion (13).

In the present study, we exploited a *Δppe38-71* deletion mutant to test whether a lack of PPE38 and its effectors will result in altered macrophage responses during infection. Initially, we verified that the strains used in this study have the same phenotype of diminished PE-PGRS protein secretion previously observed (13), as well as verifying the presence and absence of PPE38 by western blot (**Figure 1A**). Next, we measured the growth of *M. tuberculosis* CDC1551, the *Δppe38-71* mutant and the complemented strain to determine if any growth differences exist. No significant differences were found between the strains over a 20-day growth period (**Figure 1B**). To reduce variability, 4-day old mycobacterial cultures were used for all infections. The growth of each strain was monitored within THP-1 macrophage-like cells for 28 hours by enumerating cell counts at various time points (4 hours, 18 hours and 28 hours) post-infection. No significant differences were found in mycobacterial proliferation or macrophage cell death associated with any of the strains over the 28-hour period (**Figure 1C**). For the mass spectrometry experiments, we omitted antibiotic treatments for the killing of extracellular bacteria, which could result in confounding factors in this experimental design. Instead, extracellular bacteria were removed by multiple washes. The number of remaining bacteria was enumerated by CFU and a two log decrease of extracellular bacteria was observed (**Figure 1D**). Mycobacterial uptake was measured by plating intracellular bacteria at 4 hours, 18 hours and 28 hours to rule out uptake of the residual extracellular mycobacteria during the incubation period. The intracellular bacterial load remained steady across the time points and no significant differences were detected, thereby indicating little to no ongoing uptake within the time frame of this experiment (**Figure 1E**). The percentage uptake from the original titre at 4 hours after infection was 10-15% for all strains, with no significant difference between strains (**Figure 1F**). Lastly, an MTT assay was used to determine macrophage viability at 18 hours post-infection. A decrease in THP-1 viability between infected and uninfected states was observed (**Figure 1G**). However, no statistically significant differences in cell death were observed in macrophages infected with the different strains (**Data Sheet 1** and **Table S1**). These experiments demonstrate similar growth of *M. tuberculosis Δppe38-71* and wild type *in vitro*. The *Δppe38-71* mutation does not alter macrophage viability compared to wild type, nor is intracellular survival of *M. tuberculosis Δppe38-71* affected compared to wild type in our experimental conditions. These experiments indicated that abrogation of PE-PGRS/PPE-MPTR secretion to the extracellular milieu does not cause the macrophage to clear the mycobacterial infection or succumb to it. However, given the immunogenic nature of the PE-PGRS and PPE-MPTR proteins some effect is to be expected upon loss of their secretion (28, 29). We therefore investigated the proteome response of THP-1 macrophage-like cells to infection with *M. tuberculosis* CDC1551, *Δppe38-71* and the complemented strain.

### Label-Free Proteomics Reveals Time-Dependent Differences in Pro-Inflammatory Responses in Macrophages When Exposed to *M. tuberculosis* Strains Lacking PPE38-and PPE38 Controlled Proteins

Whole-cell lysates were harvested from infected macrophages at 4 hours and 18 hours post-infection and analysed by label-free mass spectrometry. A total of 2052 confident protein groups (FDR < 1%) were identified after filtering for two unique peptides and a minimum of two values per replicate in at least one of the groups. The label-free quantification (LFQ) algorithm in MaxQuant was used for relative protein quantification, and no differences in the mean distribution of intensity values were observed (**Supplementary Figure S1A**). Principal component analysis (PCA) of LFQ intensities was used to determine clustering within and between replicates of each test group at each time point. No distinct clustering was observed at the 4 hours time point (**Supplementary Figure S1B**), indicating that early innate immune responses are similar between macrophages infected with the different strains. However, a clear separation of the groups could be observed at the 18-hour time point in the first component, indicating that the majority of the variation is due to strain-specific features (**Supplementary Figure S1C**). The same analysis of the temporal macrophage responses within the groups at different time points also revealed separation in the first component (**Supplementary Figures S2D–F**).

Next, we determined the differentially regulated proteins in the infected THP-1 macrophage-like cells. There was no significant difference in protein expression between *M. tuberculosis* CDC1551 and *Δppe38-71* infected macrophages at 4 hours post-infection (**Figure 2A**). At 18 hours post-infection, a total of 39 proteins were downregulated in macrophages infected with *M. tuberculosis Δppe38-71* compared to *M. tuberculosis* CDC1551, while 11 proteins were upregulated (**Figure 2B**). Furthermore, macrophages infected with *M. tuberculosis* CDC1551 had an increased temporal cytokine expression profile, where the majority of the 39 upregulated proteins were involved in the expression of proteins associated with a pro-inflammatory response (**Figures 2C, G**). In contrast, *M. tuberculosis Δppe38-71-*infected macrophages had a remarkably low pro-inflammatory response to infection relative to wild type-infected macrophages (**Figures 2D, G**). Macrophages infected with the complemented strain displayed largely a similar temporal profile as those infected with *M. tuberculosis Δppe38-71* (**Figures 2E, F**). This may be due a number of reasons, the most likely candidates involving the promotor or the physiological state of the bacteria upon infection. As the hsp60 promotor is constitutively expressed and at high amounts it is unlikely that the lack of complementation in the macrophage is due to a lack of protein products from the *ppe38-71* operon (31). We do indeed observe greater variability within the replicates of the complement strain which likely results in reporting of non-significant results from hypothesis testing (**Supplementary Figures S1C, F**). Finally the PE-PGRS proteins are cell surface proteins and the presence of detergent is known to influence the mycobacterial capsule (9, 32, 33). Thus, the physiological state of the bacteria may be altered due to the presence of detergent in the culture media prior to infection. To gain more insight, we examined the expression of individual proteins and observed at least partial complementation in, among others, interleukin 1 Beta (IL-1B), nuclear factor kappa B (NF-kB) 2 and ‘-5’-oligoadenylate synthase 2 (OAS2) as indicated by individual fold change values (**Figure 2G**, **Data Sheet 1** and**Table S2**). Lastly, gene set enrichment analysis of the differential response at 18 hours post infection between *M. tuberculosis* CDC1551 and *Δppe38-71-*infected macrophages indicated NF-kB signalling as the most enriched pathway (**Supplementary Figure S2A**). Furthermore, key proteins that were differentially regulated during infection with these strains included IL-1B (**Supplementary Figure S2B**), NF-kB 2 (**Supplementary Figure S2C**), retinoic acid-inducible gene I (Rig-I/DDX58) (**Supplementary Figure S2D**) and Interferon stimulated gene 15 (ISG15) (**Supplementary Figure S2E**), indicative of both altered interleukin and interferon responses, all of which can be controlled by NF-kB signalling.

While we could not definitively state that the major response observed here is driven by the *ppe38-71* deletion, we do observe distinct trends in certain key proteins associated with an inflammatory response. These observations suggest a role for NF-kB signalling based on enrichment analysis of the significantly regulated proteins, which may shed light on the low inflammatory response of the macrophage to *M. tuberculosis Δppe38-71*. Initiating an inflammatory response is an energy-intensive process and requires upregulation of multiple target proteins and thus increased transcription and translation. To maintain a balance in the proteome, proteins need to be degraded in relation to this increased synthesis (34–36). This phenomenon is known as protein turnover. To further corroborate our findings, the protein turnover of macrophages infected with the different strains, as well as an uninfected control was determined.

### Proteostasis Is Affected by the Presence of PPE38-Controlled Proteins

Pulse-chase stable isotope labelling by amino acids in cell culture (pSILAC) can be used to determine protein turnover, and thus proteostasis, of an organism by mass spectrometry (25, 37, 38). Terminally differentiated THP-1 cells are unable to replicate and thus do not naturally dilute the proteins by division, providing a useful model for studying protein homeostasis. THP-1 macrophage-like cells infected with the *M. tuberculosis* CDC1551, *Δppe38-71* and complemented strains, as well as control uninfected macrophages, were sampled at multiple time points to determine protein turnover in response to infection (**Supplementary Figure S3A**). A total of 1257 protein half-lives were calculated across all four conditions, after filtering for two unique peptides, the presence of a heavy/light ratio in at least three time points and a coefficient of determination (R^2^) greater than 0.85 (**Data Sheet 1** and**Table S3**).

The percentage of heavy amino acid (lysine and arginine) incorporation increased linearly over time to a maximum of 30% incorporation in all conditions (**Supplementary Figure S3B**). Interestingly, no shift in the distribution of protein half-lives occurred between any of the conditions (**Supplementary Figure S3C**). Half-lives were highly correlated within the conditions with coefficients of determination (R^2^) values found to be 0.92 between *M. tuberculosis Δppe38-71* and *M. tuberculosis* CDC1551-infected macrophages (**Supplementary Figure S3D**), and between macrophages infected with *M. tuberculosis* CDC1551 or the complemented strain (**Supplementary Figure S3E**). The R^2^ values of half-lives from uninfected macrophages compared to *M. tuberculosis* CDC1551 were only marginally less than that of the uninfected counterparts (**Supplementary Figure S3F**). This was surprising, as it was expected that less correlation would be observed between infected and uninfected macrophages due to increased protein synthesis during infection.

As no overall differences were observed when analysing global protein turnover rates, we used hierarchical clustering to assign proteins with correlated half-lives to distinct clusters. The protein turnover profiles between the uninfected cells and *M. tuberculosis* CDC1551*-*infected macrophages showed the greatest differences (**Figure 3A**). The individual proteins grouped into two distinct clusters, where shifts in the marginal means were primarily driven by *M. tuberculosis* CDC1551 (**Figures 3B, C**). Deviations of marginal means, the mean half-life of each protein from each group, in response to infection reflect similar trends to those observed in the protein expression profiles. The response to infection with *M. tuberculosis* CDC1551 compared to the uninfected control acts as the driver that separates cluster 1 and 2. While infection by the *M. tuberculosis Δppe38-71* strain has an effect on the protein half-lives compared to uninfected, it is not as pronounced as that observed for wild type. This is in line with our protein expression results where a modest change was observed during infection. Furthermore, these clusters represent either an increase (cluster 1) or decrease (cluster 2) in protein half-lives and these clusters were used for pathway enrichments. The enrichments indicate that half-lives associated with cluster 1 are involved in proteome integrity and maintenance, while those associated with cluster 2 are involved in the inflammatory response (**Figure 3D**). Taken together, macrophages infected with *M. tuberculosis* CDC1551 induce robust inflammatory responses, with dynamic shifts in both expression and half-life. However, these effects are less pronounced when challenged with *M. tuberculosis Δppe38-71*. As in our other proteomics experiments, the complemented strain clustered closer with *M. tuberculosis Δppe38-71* infections than infection by CDC1551 and all infections were distinct from the uninfected profile.

### Protein Half-Life Differences Between *M. tuberculosis* CDC1551- and *Δppe38-71-*Infected Macrophages Point to an Altered Pro-Inflammatory Response

To further investigate the effect of protein half-lives of infected macrophages, the differential turnover of individual proteins was assessed using hypothesis testing. The protein half-lives were filtered prior to statistical testing to remove all missing values from the test groups. From these tests, we identified 24 proteins with differentially regulated half-lives between *M. tuberculosis* CDC1551 and *M. tuberculosis Δppe38-71* (**Figure 4A**) as well as 10 differentially regulated half-lives between *M. tuberculosis* CDC1551 and macrophages infected with the complemented strain (**Figure 4B**). Of these proteins, 17 were unique to macrophages infected with *M. tuberculosis Δppe38-71* compared to *M. tuberculosis* CDC1551-infected macrophages, and three were unique to macrophages infected with the complemented strain compared to *M. tuberculosis* CDC1551-infected macrophages. Seven proteins were shared between these conditions (**Figure 4C**). While some proteins are complemented, a similar trend as with the label-free comparisons is observed (**Data Sheet 1** and**Table S4**).

The proteins displaying differential half-lives in the *M. tuberculosis Δppe38-71* infected compared to *M. tuberculosis* CDC1551-infected macrophages were split into upregulated (slower turnover than in *M. tuberculosis* CDC1551-infected macrophages) and downregulated proteins (faster turnover). These proteins were used for KEGG pathway analysis and showed enrichment of fatty acid degradation pathways in the slower protein turnover cluster. Interestingly, Th1/Th2 cell differentiation was enriched in the faster turnover cluster (**Figure 4D**). From the hypothesis test, a number of immune-related proteins displayed differential half-lives. Proteins such as PSMA4, Stat-1 and Tapasin had a rapid turnover in *M. tuberculosis* CDC1551-infected macrophages compared to the other conditions (**Supplementary Figure S4A**). These proteins are involved in MHC class I presentation in the case of PSMA4 and Tapasin, or inflammatory cell signalling which is mediated by Stat-1. Interestingly, modulation of the MHC class I antigen presentation pathway by PPE38 has been demonstrated previously (39). Furthermore, proteins affecting innate immunity with slow turnover in *M. tuberculosis* CDC1551-infected macrophages included Glyoxalase I and Leukosialin (**Supplementary Figure S4B**). The glyoxalases are used for the detoxification of α-oxaldehydes, in particular methylglyoxal, in eukaryotic cells (40–42). Increased methylglyoxal can be produced by excess glucose or lack of phosphates by interfering with the glycolysis pathway and lead to the production of advanced glycation endproducts (AGE) (43). In turn AGE can stimulate the NF-kB pathway resulting in M1 polarization of the macrophage (44). As we see a clear inflammatory response in CDC1551-infected macrophages we expect an increase in the half-life of glyoxalase in order to cope with the detoxification of MG. Likewise, Leukosialin (CD43) is a cell surface protein that has been shown to interact with *M. tuberculosis* Cpn60.2 to induce TNF-α production (45), however, its expression can also commit T-cells to Th1 a response (46, 47).

Taken together, there is a robust macrophage response to infection with *M. tuberculosis* CDC1551 while little to no response is observed when challenged with *Δppe38-71.* Mixed results were observed for macrophages infected with the complemented strain thus obscuring the effect and requiring further investigation into the root cause of this differential phenotype. Based on our observations thus far it is certain that infection by the *M. tuberculosis* CDC1551 strain is stimulating the pro-inflammatory pathways of THP-1 macrophage-like cells. This is the expected response to infection by bacteria; however this result is not observed to the same extent when challenged with *M. tuberculosis Δppe38-71*. The loss of PPE38 controlled PE-PGRS proteins eliminate a number of PAMPs which may explain this observation, however the complementation does not fully restore the macrophage responses, which obscures the result.

### *M. tuberculosis Δppe38-71* Is Only Partially Complemented *In Vitro* and Complementation Is Detergent Dependent

The mixed response observed in the THP-1 macrophage-like cells infected with the complemented strain prompted us to further investigate possible differences between the bacilli under different growth conditions. For this, we analysed the proteome and secretome profiles of the different *M. tuberculosis* strains *in vitro*. As shown above, the PE-PGRS secretion phenotype is complemented at day four of *in vitro* growth (**Figure 1A**) and there are no significant differences in optical density at this time point (**Figure 1B**). We, therefore, sampled whole-cell lysates and supernatants from four-day-old cultures, which reflects the state of the strains used for infection. As PE-PGRS proteins are associated with the cell surface and thus susceptible to detergents, we removed Tween-80 by sequential washing and allowed all three strains to grow for an additional four days to represent a detergent-free supernatant. We found no clear separation between strains from the whole-cell lysate fraction (**Figure 5A**) in the first principal component, thus indicating minimal global variation between strains. However, separation in the first principal component could be observed in supernatant samples from mycobacteria cultured in Tween-80, and the complemented strain clusters with the *Δppe38-71* strain (**Figure 5B**). Interestingly, the secretome profile of the complemented strain clusters closer to *M. tuberculosis* CDC1551 when detergent is omitted from the culture supernatants (**Figure 5C**). Western blots of all three fractions were used to determine the localisation and presence of PE-PGRS proteins in these samples. We found the majority of PE-PGRS proteins in the supernatants containing detergent (detergent fraction) and no PE-PGRS proteins in the detergent-free supernatants (**Figure 5D**).

Comparison of differential protein expression in the whole-cell lysates between *M. tuberculosis* CDC1551 and *M. tuberculosis Δppe38-71* or the complemented strain revealed only two differentially regulated proteins below a q-value cut off set to 0.05, one of which was PPE71 (**Data Sheet 1** and**Table S5**). However, when comparing the detergent fraction of *M. tuberculosis Δppe38-71* to *M. tuberculosis* CDC1551, downregulation of multiple PE/PPE proteins was observed (**Figure 5E**, **Data Sheet 1** and**Table S6**). Complementation of this phenotype was observed, but partial, as many of the PE/PPE proteins still showed significantly different protein abundance relative to *M. tuberculosis* CDC1551 (**Figure 5F**, **Data Sheet 1** and**Table S6**). Finally, comparisons of the detergent free fraction of *M. tuberculosis Δppe38-71* to *M. tuberculosis* CDC1551 had a total of 142 differentially regulated proteins, 22 of which were downregulated and 120 were upregulated (**Supplementary Figure S5A**, **Data Sheet 1** and**Table S7**). Complementation in the detergent-free fraction was much more pronounced with 7 proteins significantly upregulated and 2 proteins significantly downregulated as compared to *M. tuberculosis* CDC1551 (**Supplementary Figure S5B**, **Data Sheet 1** and**Table S7**). However, the PE/PPE proteins did not feature prominently within this fraction and are likely associated with the cell surface.

These results show that the PE-PGRS and PPE-MPTR proteins are indeed expressed in the complemented strain as indicated in **Figure 1A**, however to a lesser extent than in wild type. As this represents the state used for infection, it is likely that the lack of full complementation stems from the culturing conditions prior to infection. To corroborate our infection proteomics findings, cell-free supernatants from detergent-free cultures (**Figures 5E, F**) were used to stimulate differentiated THP-1 macrophage-like cells. As pro-IL-1B was the most differentially regulated protein, we probed for expression of this protein by Western blot 18 hours after stimulation and found similar differential regulation as observed in our infection proteomics data (**Supplementary Figure S5C**). Macrophages stimulated with *M. tuberculosis* CDC1551 supernatants displayed a significantly higher IL-1B expression compared to *Δppe38-71*, and this phenotype was partially complemented (**Supplementary Figures S5D, E**).

By analysing the spatial distribution of proteins in *M. tuberculosis* cultures at their metabolic state before infection, we could show that complementation of PE-PGRS secretion was not fully restored prior to infection in the complemented strain (**Figures 5E, F**). However, the PE-PGRS secretion can be restored in the complemented strain when detergent is omitted, which is likely due to the surface localisation of PE/PPE proteins that are detached in the presence of detergent. By stimulating macrophages with cell-free supernatants, representing a high or low abundance of PE/PPE proteins, we could show differential regulation of IL-1B similar to infection with live *M. tuberculosis* CDC1551 and *Δppe38-71* strains. As seen in **Figure 5**, the composition of the *M. tuberculosis Δppe38-71* secretome relative to *M. tuberculosis* CDC1551 is a complex mixture of differentially secreted proteins not only limited to PE/PPE proteins. However, the most down-regulated proteins are indeed PE/PPE proteins, with members from both the PE-PGRS and PPE-MPTR sub-families (**Figure 5E** and **Data Sheet 1: Table S6**). It is thus likely that our observations are driven not by PPE38 alone but rather a physiological state, created by the absence of PPE38, where a group of PE/PPE proteins are acting as the effectors of PPE38 and mediating a response.

### Both RelB and NF-kB p50 Are Translocated to the Nucleus of *M. tuberculosis Δppe38-71* Infected Macrophages

NF-kB signalling regulates inflammatory responses in innate immune cells after receptor engagement with PAMPs. NF-kB subunits can be localised in the cytosol as inactive transcription factors or can be activated and translocated to the nucleus (**Figure 6A**) (48). We further investigated both NF-kB1 and NF-kB2, representing canonical and non-canonical NF-kB signalling pathways, respectively. The canonical NF-kB1 p105 and p50 subunits were detected in all tested conditions (**Figure 6B**, NF-kB1). Little to no cleavage of NF-kB2 was observed at 18 hours post-infection in any of the conditions (**Figure 6B**, NF-kB2 p52). Upregulation of the p100 subunit and pro-IL-1B (**Figure 6B**, NF-kB2 p52; IL-1B) was observed for *M. tuberculosis* CDC1551-infected macrophages. This is congruent with our other observations and indicative of a pro-inflammatory response in CDC1551-infected macrophages (49). Furthermore, our proteomics results suggest an increased interferon signalling in *M. tuberculosis* CDC1551-infected macrophages. We used ISG15 as our downstream target to validate these results and indeed found upregulation of ISG15 in *M. tuberculosis* CDC1551-infected macrophages at 18 hours compared to other conditions. We did not directly observe the STING pathway in the proteomics results, but did observe evidence for RIG-I induction. However, the interferon pathway is reportedly stimulated by STING in macrophages infected with *M. tuberculosis* (50). We therefore used Western blotting to validate expression levels of this receptor and found no differences in RIG-I expression in any of the infection conditions (**Supplementary Figure S6**). This suggests that the RIG-I is not responsible for the differential ISG15 expression, which is in line with other observations (50).

Based on these results, *M. tuberculosis* CDC1551 is triggering the canonical inflammatory NF-kB pathway. Activation of NF-kB1 and/or NF-kB2 results in the translocation of either RelA or RelB, respectively. These Rel proteins act as the effectors of this pathway and initiate transcription of target genes, depending on the required immune response. Previous studies have shown increased NF-kB1 p50 levels in the nucleus of murine macrophages infected with wild type *M. marinum* compared to a *ppe38* transposon mutant (17). While a different technique and model organism was used, this result is in line with our observations. However, it remains unclear whether *M. tuberculosis Δppe38-*71 reduces or entirely inhibits canonical NF-kB signalling or whether it induces an alternative signalling pathway.

To further investigate the activation of NF-kB signalling in infected macrophages, we used confocal microscopy to determine RelA and RelB nuclear translocation in infected macrophages. RelA translocation was observed in macrophages infected with *M. tuberculosis* CDC1551 and in macrophage infected with the complemented strain after 18 hours of infection (**Figures 7A, C**). However, in contrast, no RelA translocation was observed for macrophages infected with the *Δppe38-71* strain (**Figure 7B**) or the uninfected control (**Supplementary Figure S7A**). This result was surprising as it was expected that the inflammatory response would be triggered by RelA translocation, although perhaps to a limited extent as compared to *M. tuberculosis* CDC1551-infected macrophages. The absence of RelA translocation in the *M. tuberculosis Δppe38-71* infected macrophages prompted us to investigate RelB translocation. While no RelB translocation was observed for *M. tuberculosis* CDC1551- infected macrophages or uninfected control (**Figure 7D** and **Supplementary Figure S7B**), RelB did indeed translocate to the nucleus in *M. tuberculosis Δppe38-71* infected macrophages (**Figure 7E**). We also observed translocation of RelB in the complemented strain-infected macrophages (**Figure 7F**), however, to a lesser extent than in *M. tuberculosis Δppe38-71* infected macrophages (**Figure 7I**). The duality of the translocation events observed for the complemented strain was an interesting observation, which could give an additional explanation as to why partial restoration of the inflammatory phenotype is observed. Macrophages infected with the complemented strain seemingly mimic both the wild type, in RelA translocation, and the *Δppe38-71* phenotype, in RelB translocation. This, in turn, may result in differential cytokine release on a per-cell basis. Finally, low transient levels of RelB translocation was observed in macrophages infected with the complemented strain (**Figure 7I**). This phenomenon is consistent with other observations and likely due to poor binding of IkB-α allowing for transient movement to the nucleus for RelB complexes (51–53).

Translocation of RelB suggests that *M. tuberculosis Δppe38-71* stimulates the non-canonical NF-kB pathway, which can cause a slow, yet persistent, inflammatory response instead of a burst response (54). However, we did not observe upregulation of the p52 non-canonical partner in *M. tuberculosis Δppe38-71* infected macrophages. Furthermore, slow and persistent inflammation should accumulate IL-1B over time, yet a downregulation of this cytokine was observed. Nonetheless, the p50 subunit was available as a potential partner for translocation. Based on our observations in both mass spectrometry, Western blots and microscopy, macrophages infected with *M. tuberculosis* CDC1551 are signalling through the canonical pathway characterised by RelA and p50 translocation. The translocation dynamics of macrophages infected with *M. tuberculosis Δppe38-71* strain is however unclear. To rule out the non-canonical pathway, macrophages were infected with *M. tuberculosis Δppe38-71* and probed for translocation of p50 and p52 at 18 hours post-infection. No translocation of the non-canonical NF-kB2 p52 subunit was observed (**Figure 7G**), while the canonical NF-kB1 p50 subunit was found to localise to the nucleus (**Figure 7H**). No noticeable translocation of either p50 or p52 was observed in the uninfected THP-1 macrophage-like cells (**Supplementary Figures S7C, D**). This indicates that infection with *M. tuberculosis Δppe38-71* causes translocation of RelB/p50 complexes (**Supplementary Figure S7E**), which has been shown to be associated with anti-inflammatory responses (55–57).

### Less IL-12p70 Is Found in Supernatants of *M. tuberculosis Δppe38-71* infected macrophages at 48 hours post-infection

Pro- and anti-inflammatory macrophage responses are characterised by the secretion of specific cytokines. Examples include the secretion of IL-12p70 as a marker for the pro-inflammatory response and IL-13 to represent the anti-inflammatory response (58). Macrophages were incubated for 48 hours to examine the delayed inflammatory response during infection with *M. tuberculosis Δppe38-71*. We found a greater abundance of IL-12p70 in *M. tuberculosis* CDC1551 and complement-infected macrophages relative to the *M. tuberculosis Δppe38-71* infected, and uninfected control macrophages (**Figure 8A**). This differential secretion pattern indicates that 48 hours post-infection the macrophages have still not launched a strong inflammatory response to the *M. tuberculosis Δppe38-71* strain as compared to *M. tuberculosis* CDC1551. There was also no significant difference in the secretion of IL-12p70 in *M. tuberculosis Δppe38-71* infected macrophages when compared to the uninfected control (**Figure 8A** and **Data Sheet I: Table S8**). In contrast to this, IL-13 displayed the opposite pattern with less IL-13 present in the supernatant of *M. tuberculosis* CDC1551 and complement-infected macrophages compared to macrophages infected with *M. tuberculosis Δppe38-71* (**Figure 8B**).

Taken together, these results indicate that the PE/PPE proteins controlled by PPE38 have an effect on modulating macrophage responses through NF-kB signalling. THP-1 macrophage-like cells infected with *M. tuberculosis* CDC1551 are exposed to substantially more PAMPs as seen in **Figure 5E** and thus a canonical RelA/NF-kB1 p50 pathway is initiated resulting in a strong pro-inflammatory response (**Figure 8C**). However, in the absence of PPE38 and its effectors, RelB/p50 translocates to the nucleus and likely dampens this response, resulting in an anti-inflammatory phenotype over time (**Figure 8D**).

## Discussion

*M. tuberculosis* can influence disease outcome by altering protective host innate immune responses. However, the effector molecules involved in host-pathogen interactions and altered host responses remain ill-defined. It was previously demonstrated that deletion of *ppe38-71* in *M. tuberculosis* resulted in increased virulence in a murine infection model (13). In this study, we investigated the role of *M. tuberculosis Δppe38-71* in the context of host-pathogen interactions to identify the mechanisms associated with increased virulence. An important observation made here is that the PE-PGRS and PPE-MPTR proteins controlled by PPE38 are responsible for driving altered macrophage responses. These proteins are unique to pathogenic mycobacteria and several authors have proposed a role for PE-PGRS and PPE-MPTR proteins in host-pathogen interactions (7).

Infection of macrophages with wild type *M. tuberculosis* CDC1551 results in increased expression of pro-inflammatory cytokines and chemokines, specifically, upregulation of pro-IL-1B, several interferon-inducible proteins (ISG15, IFIT1, IFIT2, IFIT3, IFIH1 and MX1) and rapid recycling of components associated with IL-12 signalling. Conversely, deletion of *ppe38-71* in *M. tuberculosis* resulted in a dampened innate immune response in infected macrophages, where IL-1B was found to be downregulated over time. This shift away from a pro-inflammatory response was observed in both temporal label-free proteomic analysis as well as protein turnover analysis using pulse SILAC labelling. However, there seems to be a disparity in the cytokine signalling of dendritic cells and macrophages when infected with ppe38 mutants in the current literature. After overnight stimulation of murine dendritic cells with *M. tuberculosis* CDC1551, *M. tuberculosis Δppe38-71* and the complemented strain there was no significant difference observed in supernatant IL-12p40/70, IL-6 and TNF-α (18). In contrast, there is a stark difference in IL-6 and TNF-α levels found in macrophages infected with the *M. marinum* wild type and a *ppe38* transposon mutant strain (16). In the latter case, TNF- α is lower in macrophages infected with a *M. marinum ppe38* transposon mutant compared to wild type at both 24 and 48 hours post infection. We did not assay for IL-6 or TNF- α, however from our cytokine assay we find less IL-12p70 in the supernatant of macrophages infected with *M. tuberculosis Δppe38-71.* Notably the difference in abundance of IL-12p70 detected in this study could easily be influenced by various factors such as cell type, MOI and timeframe in comparison to the levels measured in dendritic cells (18).

Macrophages challenged with concentrated supernatants from each strain showed the same regulation of IL-1B as during live mycobacterial infections. Here it was demonstrated that the deletion of the *ppe38-71* operon has farther reaching consequences to the cell than only this protein or even the PE-PGRS proteins. It is thus likely not one single protein is driving the macrophage response but a conglomerate of proteins representing a physiological state upon deletion of the *ppe38-71* operon. Many of the PE/PPE proteins have unknown functions, however, multiple studies have shown that these proteins are upregulated during macrophage infection (59–61) and are highly immunogenic (62). In **Figure 5E** several PE/PPE proteins were identified as being controlled by PPE38 and these are the likely candidates for intracellular effector proteins. Notably, PPE10 was represented in this cluster and has been implicated in the disruption of *M. marinum* capsule integrity, altering colony morphology and attenuation of virulence in zebrafish (63). As PPE10 was one of the most downregulated proteins in the *M. tuberculosis Δppe38-71* strain, it is likely similar phenotypes can be expected from PPE38 knock outs. However, no morphology differences were visible in *M. tuberculosis Δppe38-71*, although the upregulated proteins in **Figure 5E** were associated with intracellular proteins. While not definitive, this gives some indication of a damaged cell wall. Interestingly, *M. marinum ppe38* transposon mutants demonstrate a visible change in colony morphology (16). It is as of yet unclear whether this is due to differential regulation of PPE10 by proxy of PPE38. Nevertheless, drawing this conclusion seems likely given the evidence. Taken together, the *in vitro* profile of *M. tuberculosis Δppe38-71* prior to infection indicates decreased levels of multiple virulence factors known to induce pro-inflammatory responses in macrophages. Based on these results it is likely not one protein that drives the effect but many proteins that are altered due to the loss of the *ppe38-71* operon.

Functional enrichments revealed altered NF-kB signalling between macrophages infected with *M. tuberculosis* CDC1551 and those infected with *M. tuberculosis Δppe38-71*. Canonical NF-kB signalling is partly responsible for the induction of pro-inflammatory responses and is characterised by the translocation of the RelA protein along with the NF-kB1 subunit in a RelA/p50 complex (49). This canonical signalling pathway is induced during infection with *M. tuberculosis* CDC1551. In contrast, infection by *M. tuberculosis Δppe38-71* stimulated the signalling of a different NF-kB pathway where RelB and p50 are translocated to the nucleus. A multi-organ inflammatory response observed in mice with a RelB^(-/-)^ knock out was aggravated in a RelB^(-/-)^/p50^(-/-)^ double knock out (55, 64). This phenotype indicates that inflammation is controlled by the RelB/p50 pathway and is likely used to limit excessive inflammation during activation of the canonical NF-kB pathway. Furthermore, a study investigating responses in dendritic cells and macrophages stimulated with LPS has shown that the RelB/p50 pathway inhibits TNF-α production by modulating the canonical pathway (57). We have shown that in the absence of PPE38, this pathway is activated in infected THP-1 macrophage-like cells, which provides a molecular mechanism that could be used by *M. tuberculosis* to drive switching of inflammatory states in macrophages during infection. In addition, differential localisation of NF-kB subunits has also been previously reported for an *M. marinum ppe38* transposon mutant as revealed by spatial proteomics (16, 17).

Based on the results reported here and by others, the secretion of PPE38-dependent proteins to the extracellular milieu, where these proteins are able to interact with host proteins, can initiate a differential inflammatory cascade. In the absence of these effectors, immune dampening is observed mediated by RelB as the likely molecular switch. Th ppe38-71 region is indeed a hotspot for evolutionary activity which includes recombination events, truncations, gene fusion formation and more recently a source for phenotype sharing as a donor for horizontal gene transfer (65–68). Thus, a natural deletion of the *ppe38-71* operon can confer an evolutionary advantage by dampening the innate immune response and possibly by providing a downstream molecular mechanism for controlling macrophage polarisation states. A bacterial mechanism to dampen macrophage responses and switch the polarisation state has been shown to be mediated by effectors of the Spi-2 secretion system in Salmonella (69). Early investigations into *M. tuberculosis* HN878, a member of the lineage 2 isolates of *M. tuberculosis*, demonstrated increased virulence associated with the failure to stimulate Th1 responses (70), similar to the observations made in this study. Interestingly, the same study found that a lack of a pro-inflammatory response was associated with an increased induction of type I interferons (70). In this study we observe increased ISG15 expression, which we speculate may be as a result of the induction of the STING pathway (50). The increased production of ISG15 may partly or wholly be caused by an increase in type I interferons elicited by the *ppe38-71* mutant (71). It was further demonstrated that the decreased inflammatory response was associated with the presence of a phenolic glycolipid on the cell surface of *M. tuberculosis* HN878 (72). This phenolic glycolipid is synthesised by an intact copy of the *pks15/1* gene found in a subset of lineage two isolates (72). Later studies investigated whether an intact *pks15/1* gene confers the same hypervirulence, low inflammatory response phenotype regardless of the genetic background. The authors found that the phenolic glycolipid can act to modulate the host cytokine response but does not directly extend to a hypervirulent phenotype (73). The authors further speculate that the phenolic glycolipid forms a part of a greater genotypic and phenotypic profile of the lineage 2 strains to confer the dampened immune response and hypervirulence (73). Interestingly, we have previously found that the *ppe38-71* deletion occurs overwhelmingly within the lineage two isolates (13, 68). Furthermore, other studies have demonstrated an increase in virulence of lineage 2 isolates with a naturally occurring *ppe38-71* deletion; this virulence was partially mitigated by the heterologous introduction of this operon (13). Taken together, it is likely that the *ppe38-71* mutation, in part, plays a role in the increased virulence associated with the lineage two isolates of *M. tuberculosis* by inducing a more permissive environment for bacterial growth during infection. This is supported by the observation that a *ppe38-71* deletion mutant showed increased bacterial load at later stages of infection in mice compared to the wild type parental strain (13). Interestingly, a recent study demonstrated a similar response in IL-1B modulation, where clinical isolates that induce lower levels of IL-1B were able to successfully evade the macrophages response through decreased inflammasome activation (74). Furthermore, isolates that were associated with severe tuberculosis in patients presented with lower cytokine responses in infected peripheral blood monocytes (74). This shows that *M. tuberculosis* is capable of modulating the inflammatory response through multiple molecular mechanisms and does so by selecting for genomic variation that results in decreased inflammatory responses and increased pathogenicity.

In conclusion, we have used complementary mass spectrometry-based approaches along with follow-up validation to elucidate the role of PPE38-controlled proteins in host-pathogen interactions. Wild type *M. tuberculosis* CDC1551 strains induced the canonical NF-kB pathway to stimulate pro-inflammatory responses in infected human macrophages, whereas in the absence of PPE38-controlled PE-PGRS and PPE-MPTR proteins the alternative RelB/p50 NF-kB pathway is induced. This results in an anti-inflammatory phenotype where the macrophages fail to launch an appreciable pro-inflammatory response. Future experiments will have to identify which PE-PGRS and/or PPE-MPTR protein plays a key role in the RelB-mediated switch between macrophage polarisation states that can influence the infectious process.

## Data Availability Statement

All raw mass spectrometry data was deposited to the ProteomeXchange consortium *via* the PRIDE partner repository under the following accessions: Mycobacterium tuberculosis whole-cell lysates (PXD020814), Mycobacterium tuberculosis Tween-80 containing secretomes (PXD020813), Mycobacterium tuberculosis tween-free secretomes (PXD021168), THP-1 label-free whole-cell lysates (PXD021167) and THP-1 SILAC labelled whole-cell lysates (PXD021166).

## Author Contributions

JG: conceptualisation, formal analysis, investigation, data curation, visualization, software, writing, funding acquisition, project administration. TH: conceptualisation, data curation, methodology, funding acquisition, supervision, writing – review and editing. CB: investigation, writing - review and editing. KS: investigation, writing - review and editing. SB: investigation. IM: methodology, resources, writing - review and editing. WB: conceptualisation, data curation, resources, supervision, project administration, funding acquisition, writing - review and editing. SS: conceptualisation, data curation, resources, supervision, project administration, funding acquisition, writing - review and editing. All authors contributed to the article and approved the submitted version.

## Funding

JG would like to acknowledge the NRF for financial support under the NRF-VU Desmond Tutu Doctoral training program and the Harry Crossley Foundation for project support. TH was supported by a South African National Research Foundation-Department of Science and Technology Innovation Postdoctoral Fellowship (SFP13071721852). SS is funded by the South African Research Chairs Initiative of the Department of Science and Technology and National Research Foundation (NRF) of South Africa, award number UID 86539. The authors acknowledge the SA MRC Centre for TB Research and DST/NRF Centre of Excellence for Biomedical Tuberculosis Research for financial support for this work.

## Disclaimer

The content is solely the responsibility of the authors and does not necessarily represent the official views of the NRF.

## Conflict of Interest

The authors declare that the research was conducted in the absence of any commercial or financial relationships that could be construed as a potential conflict of interest.

The reviewer RB has declared a past collaboration with one of the authors WB at the time of review.
