# Supplementary material for: PPE38-Secretion-Dependent Proteins of M. tuberculosis Alter NF-kB Signalling and Inflammatory Responses in Macrophages
Source: Front Immunol. 2021 Jul 2;12:702359. doi: 10.3389/fimmu.2021.702359 (PMC8284050; doi:10.3389/fimmu.2021.702359)
Supplement: Supplementary file 1 [file DataSheet_1.pdf]

Supplemental Figures

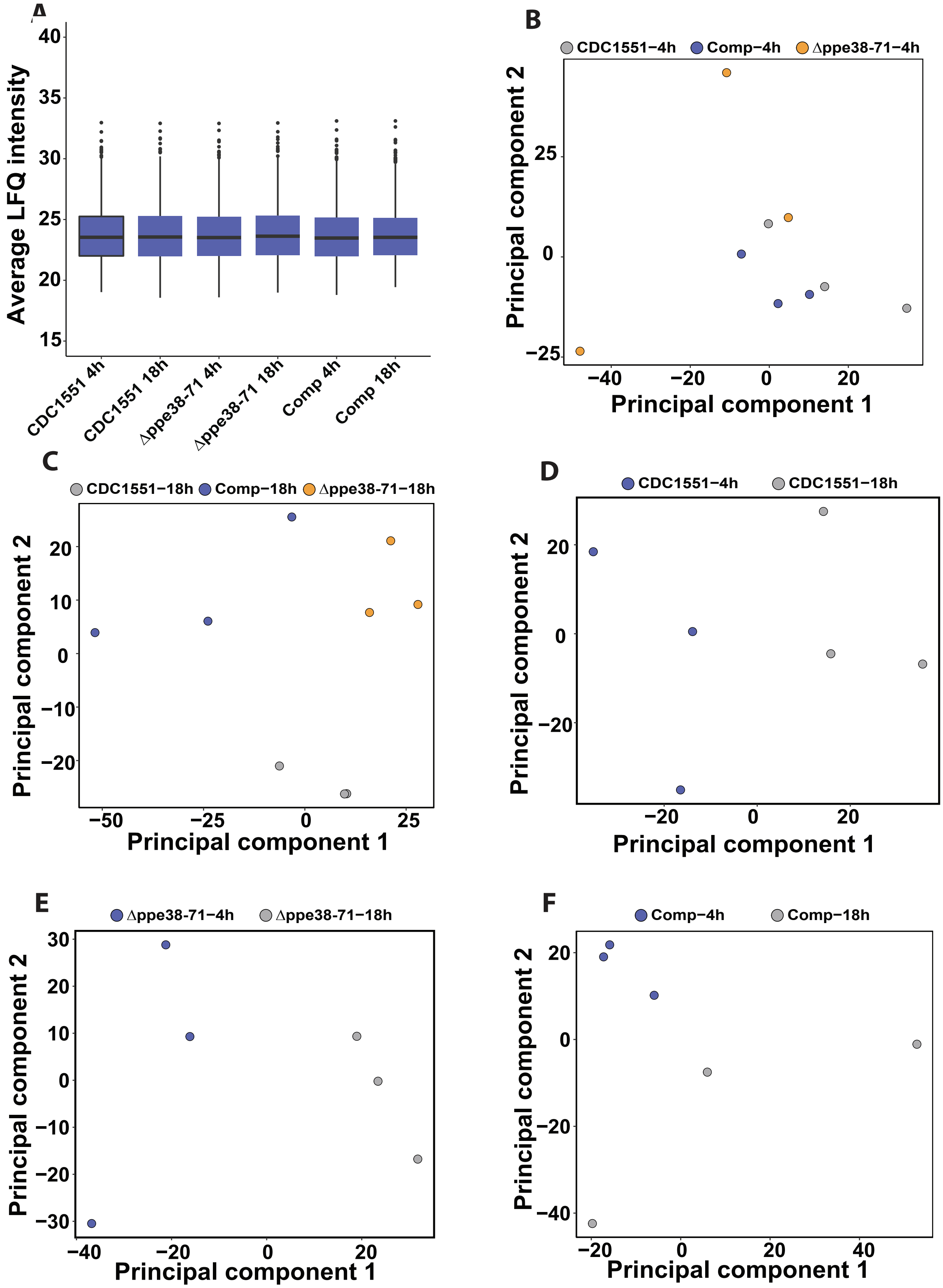

Figure S1: Quality control of label-free mass spectrometry data, related to figure 2: A) Boxplot representing LFQ intensities across all samples. Significant deviations from the mean between samples were ruled out by hypothesis testing using one-way ANOVA and Tukey HSI post-hoc testing, q-value was set at 0.05. **B)** Principal component analysis was used to assess the clustering of groups and replicates. Little to no clear separation occurs at 4h post-infection between all groups. **C)** Separation was observed between all three groups at 18h post-infection and clustered based on strain genotype. Separation occurs between *M. tuberculosis* CDC1551-infected macrophages at 4h and 18h post-infection on the first component while replicates separated on the second component in all three strains namely, **D)** *M. tuberculosis* CDC1551, **E)** Δppe38-71 and **F)** complement-infected macrophages. Data used for all samples is represented by 3 biological replicates per sample.

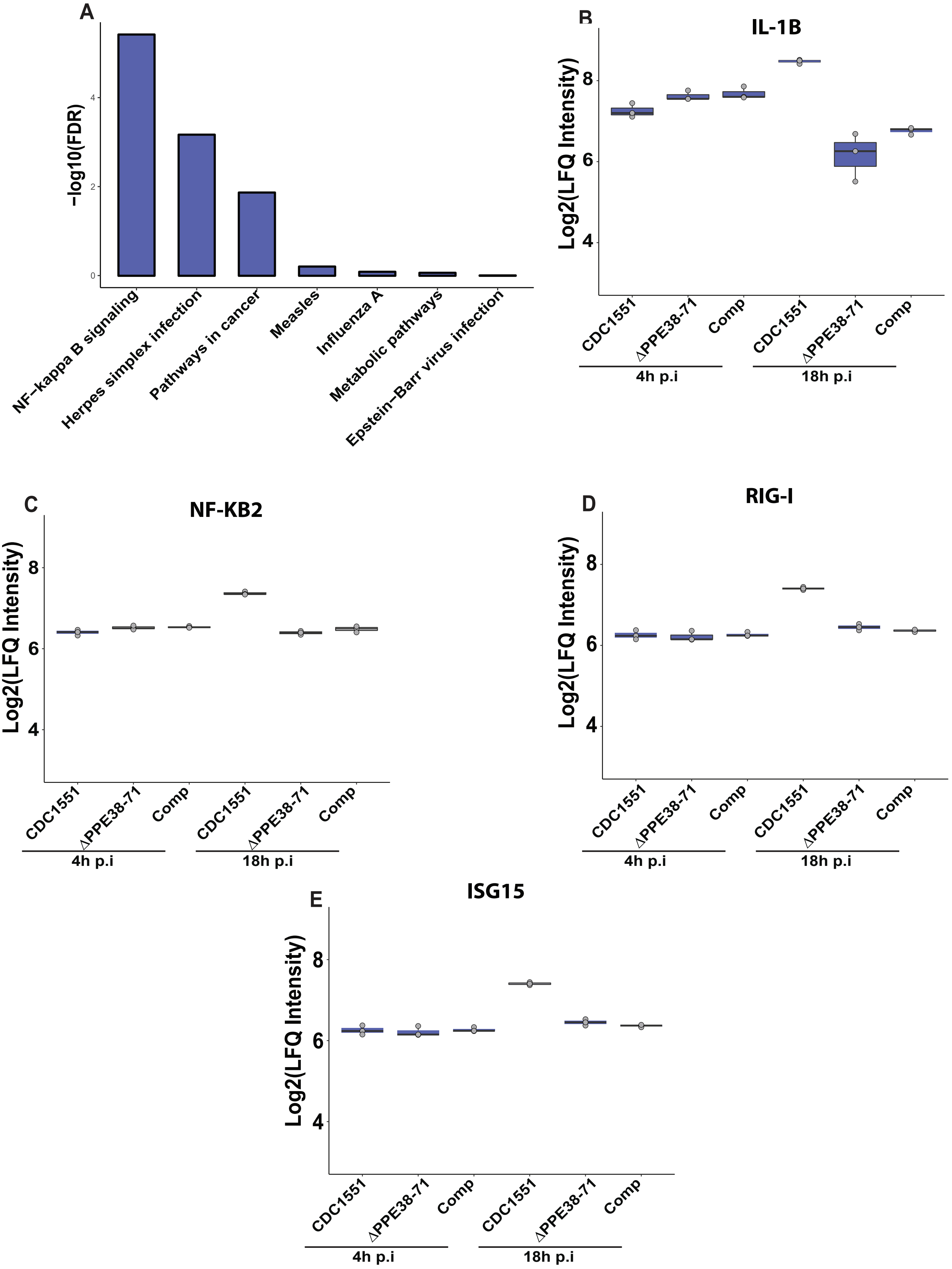

Figure S2: Enrichment analysis indicates altered inflammatory responses in *M. tuberculosis*-infected macrophages, related to figure 2. A) Gene ontology enrichment using the WebGestalt server. Significantly regulated proteins and their respective effect sizes, as determined in Figure 3, were used as the gene set input and enriched against the Kyoto Encyclopaedia of Genes and Genomes and scaled using the false-discovery rate (FDR). Log2 LFQ intensities for **B)** IL-1B, **C)** NFkB2, **D)** RIG-I and **E)** ISG15 identified from macrophages infected with the different strains at the indicated time points. Individual points in each group represent each biological replicate, while error bars represent the standard deviation.

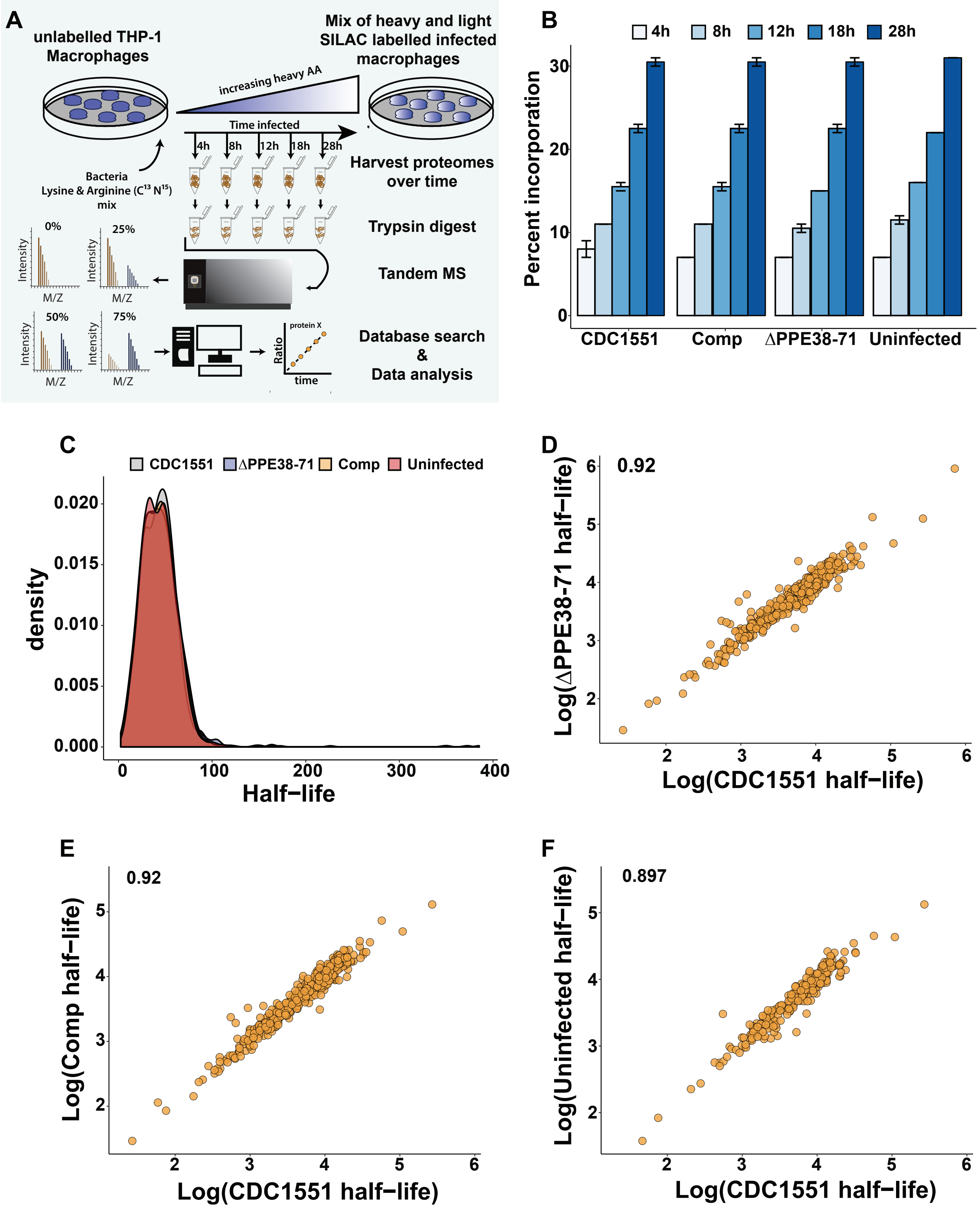

Figure S3. Global protein turnover in THP-1 macrophages is not significantly altered during infection, related to figure 3. A) A pulse SILAC labelling approach was used to obtain protein half-lives. Proteins were harvested at 4h, 8h, 12h, 18h and 28h post-infection from uninfected THP-1 macrophages as well as macrophages infected with *M. tuberculosis* CDC1551, *M. tuberculosis Δppe38-71* and the complemented strain.
B) Incorporation of "heavy" arginine and lysine over time. Maximal incorporation of ~30% was achieved in all infected macrophages and in the uninfected control. **C)** Density plot representing the distribution of protein half-lives from both infected and uninfected macrophages. Half-lives were calculated from the mean raw H/L ratios of two independent experiments. **D-F)** Multiple scatterplots of pairwise comparisons between conditions. The points of the scatter plot represent from log2 transformed half-lives, with Pearson correlation coefficients for each comparison.

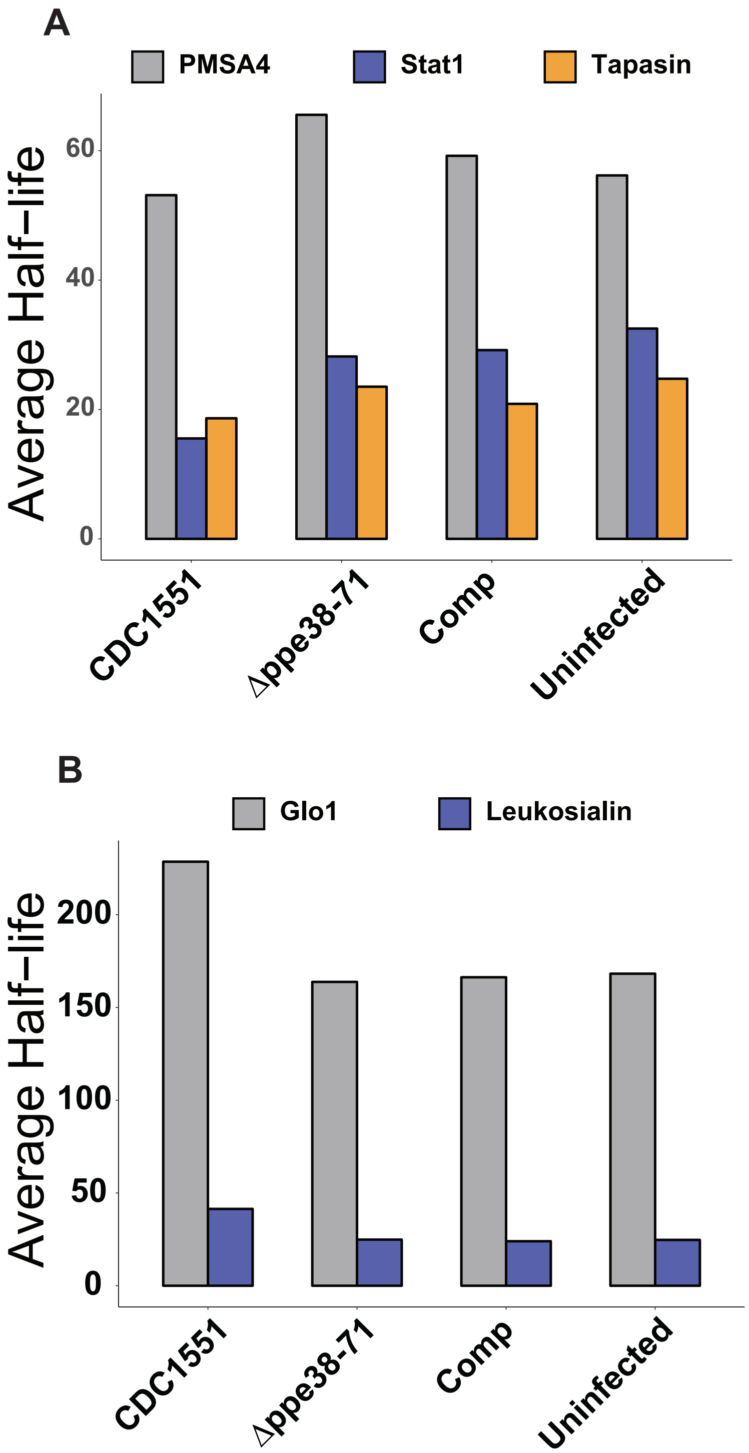

Figure S4: Proteins involved in response to infection that have differentially regulated half-lives between THP-1 macrophages infected with *M. tuberculosis* CDC1551 and *M. tuberculosis* Δppe38-71, related to figure 4:

From the differentially regulated half-lives, selected proteins with a **A)** rapid or **B)** slow turnover in THP-1 macrophages infected with *M. tuberculosis* CDC 1551 compared to other conditions are displayed. Values are representative of two independent pSILAC experiments.

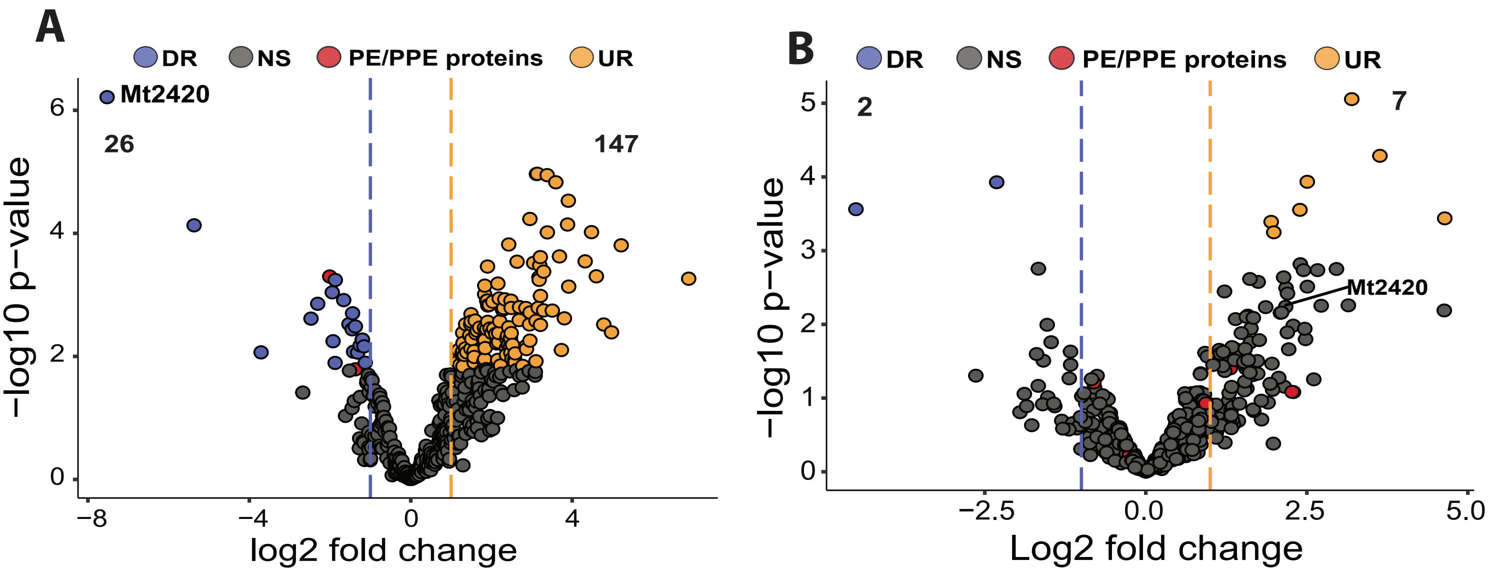

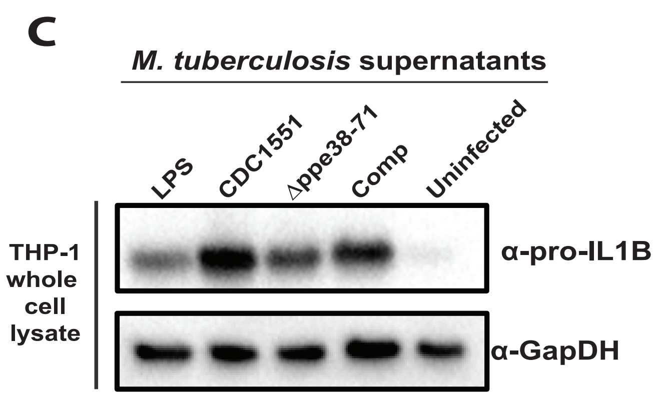

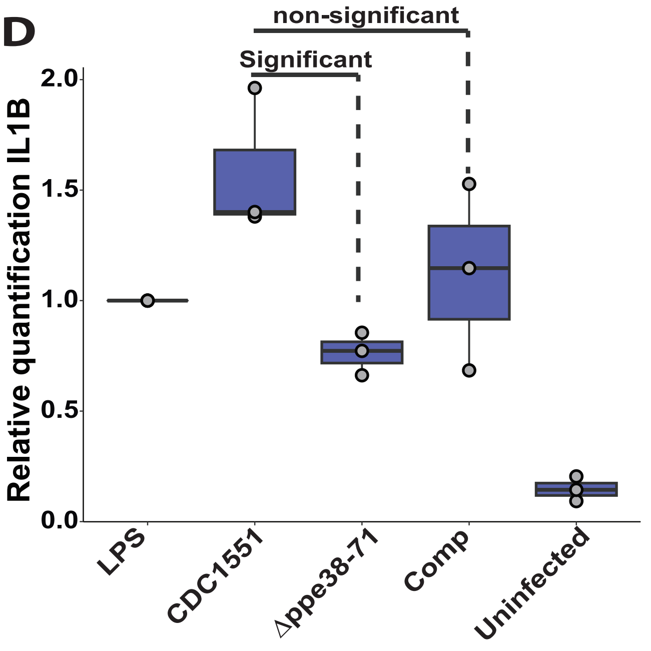

E One way Anova + Tukey HSD posthoc

Strain	diff	lwr	upr	p.adj
Comp-CDC1551	-0.46	-1.12	0.20	0.22
Δppe38-71-CDC1551	-0.82	-1.48	-0.16	0.01
LPS-CDC1551	-0.58	-1.24	0.08	0.09
Uninfected-CDC1551	-1.43	-2.09	-0.78	0.00
dPPE38-Comp	-0.36	-1.01	0.30	0.43
LPS-Comp	-0.12	-0.78	0.54	0.97
Uninfected-Comp	-0.97	-1.63	-0.31	0.00
LPS-Δppe38-71	0.24	-0.42	0.90	0.76
Uninfected-Δppe38-71	-0.62	-1.27	0.04	0.07
Uninfected-LPS	-0.85	-1.51	-0.19	0.01

Figure S5: Label-free mass spectrometry analysis of detergent free *M. tuberculosis* supernatants shows restored complementation but no PE/PPE proteins, related to figure 5.

Volcano plots depicting differential expression of proteins found in the detergent-free supernatants from **A)** *M. tuberculosis* Δppe38-71 and **B)** complemented strain compared to *M. tuberculosis* CDC1551. A q-value cut-off was set to 0.05 and the log fold change was set to 1. Data is representative of three independent experiments. THP-1 macrophages were stimulated with cell-free supernatants from *M. tuberculosis* CDC1551, *Δppe38-71* or complemented strains. Stimulation with lipopolysaccharides from *E. coli* served as a positive control and unstimulated macrophages as negative control. Macrophages were lysed and probed for IL-1B expression using **C)** Western blot, which was quantified by **D)** densitometry. The data is a representative of three independent experiments and statistical significance was determined using **E)** one-way ANOVA, followed by a Tukey HSD post-hoc test with a q-value set at 0.05.

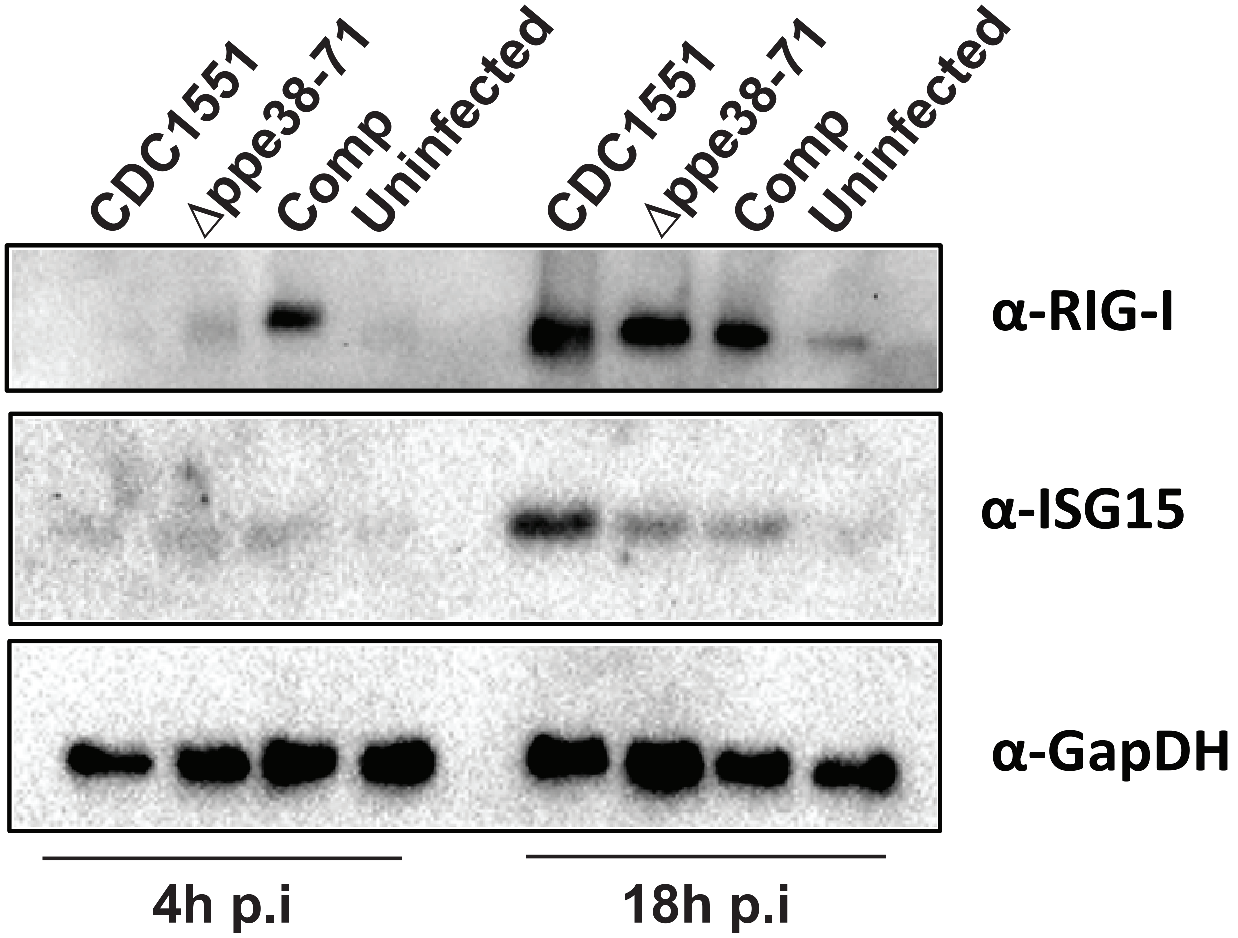

Figure S6: Expression of ISG15 and RIG-I at 18 hours post infection shows no differential regulation in RIG-I but upregulation of ISG15 in *M. tuberculosis* CDC1551, related to figure 6. THP-1 macrophages were infected with *M. tuberculosis* CDC1551, *M. tuberculosis Δppe38-71* and the complemented strain and probed for RIG-I and ISG15 at 4 hours and 18 hours post infection by Western blot. GapDH was used as a loading control and uninfected macrophages were used as a stimulus control.

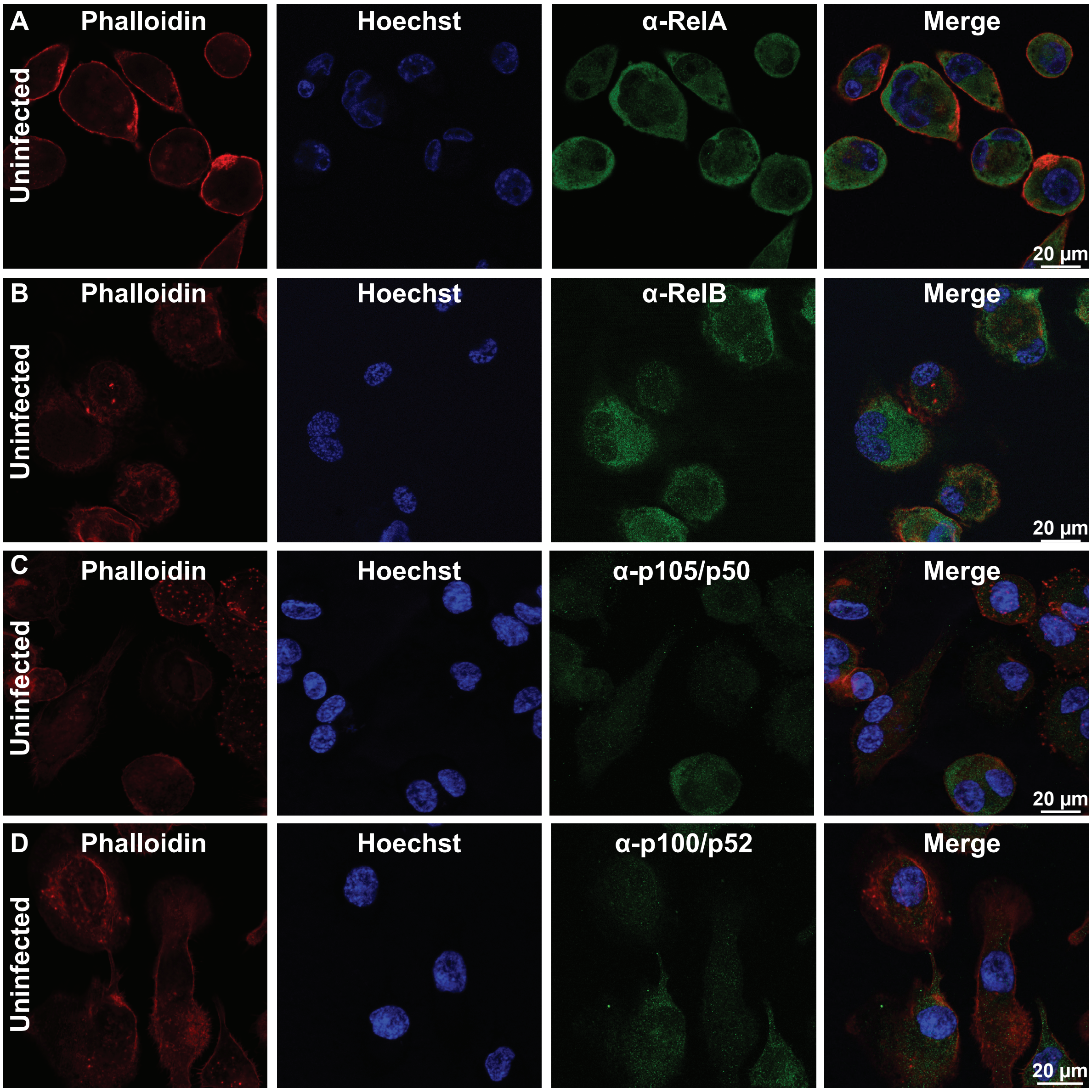

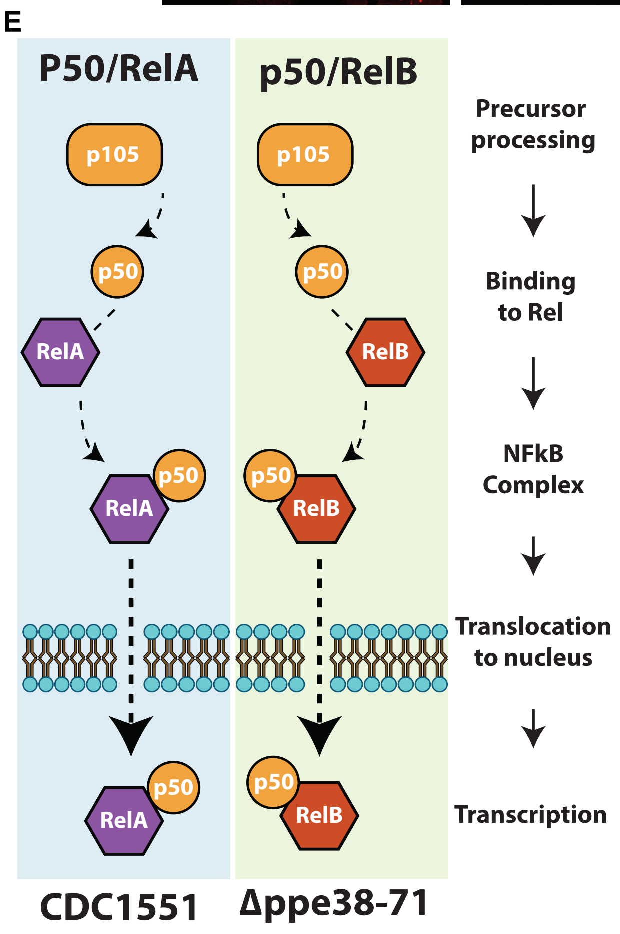

Figure S7: Confocal microscopy of RelA and RelB in control samples, related to figure 7.
Representative images of uninfected THP-1 macrophages used as control in for the experiment depicted in Figure 7. Uninfected macrophages were labelled with phalloidin (F-actin), the nucleus was stained with Hoechst and **A)** RelA, **B)** RelB, **C)** p105/p50 and **D)** p100/p52 conjugated-Alexafluor 488 was used to detect NFkB proteins. **E)** Schematic illustration of the NF-KB pathway stimulated by infection with *M. tuberculosis* CDC1551 and *M. tuberculosis Δppe38-71*.

Supplementary tables

Table S1: THP-1 MTT assay 18 hours p.i. one way ANOVA with Tukey HSD post-hoc test, related to figure 1G

Tukey's multiple comparisons test	Mean Diff.	95.00% CI of diff.	Significant?	Summary	Adjusted P Value
CDC1551 vs. dPPE38	- 0,206778	-1.94640167824652 to 1.53284634491319	No	ns	0,979930098
CDC1551 vs. Complement	0,032533	-1.70709067824652 to 1.77215734491319	No	ns	0,999916026
CDC1551 vs. Uninfected	-2,441	-4.18062401157986 to - 0.701375988420144	Yes	**	0,008714893
dPPE38 vs. Complement	0,239311	-1.50031301157986 to 1.97893501157986	No	ns	0,969639075
dPPE38 vs. Uninfected	- 2,234222	-3.97384634491319 to - 0.494598321753478	Yes	*	0,014325632
Complement vs. Uninfected	- 2,473533	-4.21315734491319 to - 0.733909321753477	Yes	**	0,008072296

Table S8: One-way ANOVA of secreted cytokines measured by ELISA. Related to figure 7

Cytokine	Comparison	p-value
IL13	Comp-CDC1551	0,9275949
IL13	Δppe38-71-CDC1551	0,0109326
IL13	Uninfected-CDC1551	0,0292327
IL13	Δppe38-71-Comp	0,0245765
IL13	Uninfected-Comp	0,0682513
IL13	Uninfected-dPPE38	0,8819416
IL12p70	Comp-CDC1551	0,9993897
IL12p70	dPPE38-CDC1551	0,0140914
IL12p70	Uninfected-CDC1551	0,0007968
IL12p70	dPPE38-Comp	0,016453
IL12p70	Uninfected-Comp	0,000899
IL12p70	Uninfected-dPPE38	0,1460341
